# Target-level convergence of directional prefrontal connectivity across Alzheimer’s disease stages: an fNIRS study

**DOI:** 10.3389/fnagi.2026.1834253

**Published:** 2026-08-24

**Authors:** Nida Mateen, Rana Muhammad Kaleem Ullah, Jisoo Baik, Keum-Shik Hong, Min-Kyoung Kang, Chang-Seok Kim, Yong-Il Shin

**Affiliations:** 1 Department of Cogno-Mechatronics Engineering, Pusan National University, Busan, Republic of Korea; 2 Biomedical Research Institute, Pusan National University Yangsan Hospital, Yangsan, Gyeongsangnam-do, Republic of Korea; 3 Institute for Future, School of Automation, Qingdao University, Qingdao, China; 4 Institute for Industrial Artificial Intelligence, School of Mechanical Engineering, Pusan National University, Busan, Republic of Korea; 5 Engineering Research Center for Color-Modulated Extra-Sensory Perception Technology, Pusan National University, Busan, Republic of Korea; 6 The Crystal Bank Research Institute, Pusan National University, Busan, Republic of Korea; 7 Department of Rehabilitation Medicine, School of Medicine, Pusan National University Yangsan Hospital, Yangsan-si, Republic of Korea

**Keywords:** Alzheimer’s disease, effective connectivity, functional near-infrared spectroscopy, mild cognitive impairment, prefrontal cortex, transfer entropy

## Abstract

**Background:**

Alzheimer’s disease (AD) is increasingly recognized as involving network-level dysfunction rather than isolated regional impairment. Although functional and effective connectivity have been studied in dementia, the reorganization of directional information flow within prefrontal networks remains incompletely characterized, particularly with functional near-infrared spectroscopy (fNIRS).

**Objective:**

This study investigated whether target-level convergence of directional effective connectivity within the prefrontal cortex differs across clinically defined cognitive groups, with emphasis on the mild cognitive impairment (MCI) vs. AD contrast.

**Methods:**

fNIRS signals were recorded from 60 participants (healthy controls (HC), MCI, and AD) during resting state and cognitive paradigms, including working memory (2-back), semantic verbal fluency (SVFT), and Stroop tasks. Signals from 48 prefrontal channels were aggregated into eight anatomically defined regions. Directional interactions were quantified using transfer entropy (TE) with adaptive hyperparameter tuning. Group differences were assessed using linear mixed-effects models with covariate adjustment and target-wise false discovery rate correction. Robustness was evaluated through parameter sensitivity, covariate, and surrogate-normalization analyses.

**Results:**

The analysis revealed structured target-level convergence patterns of directional connectivity across clinically defined cognitive groups, with the most prominent alterations observed in the MCI vs. AD comparison. Compared with MCI, AD showed reduced directed information flow to the right frontopolar cortex (FPC-R) during the 2-back and SVFT tasks (*β* = −0.71 to −1.18, *q* < 0.05), indicating diminished convergence of inputs from distributed prefrontal regions. Conversely, convergence toward the left dorsolateral prefrontal cortex (DLPFC-L) increased across multiple paradigms (*β* = 0.56–0.85, *q* < 0.05). Additional task-specific alterations were observed during the Stroop paradigm, although some effects demonstrated reduced stability in sensitivity analyses. Exploratory ROC analysis suggested contrast-specific discriminative utility of selected convergence features, with moderate performance in MCI vs. AD (AUC ≈ 0.77).

**Conclusion:**

These findings suggest that MCI and AD are associated with distinct patterns of directional information flow within prefrontal networks, characterized by reduced convergence toward FPC-R and increased directional influence toward DLPFC-L. Collectively, the results indicate that TE applied to task-based fNIRS can capture directional alterations in network connectivity, highlighting the potential of directional connectivity metrics for studying network-level changes associated with cognitive decline.

## 1 Introduction

Alzheimer’s disease (AD) is the leading cause of dementia worldwide, accounting for almost two-thirds of cases (Kamatham et al., 2024). The disease is characterized by progressive cognitive decline affecting memory, executive function, and semantic processing, accompanied by widespread synaptic and network-level pathology (Serrano-Pozo et al., 2011; DeTure and Dickson, 2019). The AD continuum is commonly described as a clinical spectrum comprising cognitively normal individuals, subjective cognitive decline (SCD), mild cognitive impairment (MCI), and clinically diagnosed AD dementia (Oikonomou et al., 2025). Within this framework, SCD represents an early symptomatic stage characterized by self-perceived cognitive decline without objective impairment on standard neuropsychological testing, whereas MCI represents a clinically and biologically heterogeneous stage characterized by measurable cognitive deficits without functional dependence (Rosenberg and Lyketsos, 2008). Clinically diagnosed AD dementia is characterized by more pronounced cognitive impairment accompanied by functional decline. Longitudinal studies show that a significant proportion of individuals with MCI progress to AD (Salemme et al., 2025), although conversion rates vary by etiology, biomarkers, and follow-up duration; some remain stable or revert to normal cognition (Petersen et al., 2014; Wilks et al., 2024). Although the present study did not include an SCD group, this heterogeneity underscores the need to identify neural network features that reflect stage-associated differences in information flow across selected clinically defined stages within the AD continuum, in addition to categorical clinical diagnosis.

AD is increasingly recognized as a disorder of brain networks rather than isolated regional dysfunction. Large-scale fMRI studies have revealed disrupted functional organization within canonical resting-state networks, including the default mode, frontoparietal control, dorsal attention, and salience networks, whereas EEG studies have shown altered electrophysiological connectivity and declining network segregation and efficiency across the AD continuum (Greicius et al., 2004; Wang et al., 2015; Dragomir et al., 2019). These network alterations are closely associated with amyloid and tau pathology (Fu et al., 2021; Mijalkov et al., 2023). They may also occur prior to overt structural atrophy in the disease course (Hu et al., 2024). However, these large-scale network approaches may not fully resolve how information is redistributed within specific cortical subsystems, such as the prefrontal cortex, that support executive and semantic processes.

The prefrontal cortex (PFC) is a functionally specialized subsystem central to executive control, working memory, decision-making, and semantic retrieval, which are cognitive domains affected early across the AD continuum (Friedman and Robbins, 2022; Clément et al., 2013). Subregions of the PFC, including the dorsolateral, ventrolateral, orbitofrontal, and frontopolar cortices, support distinct but interacting cognitive functions (O'Reilly, 2010; Burgess et al., 2007; Kringelbach, 2005). Neurodegenerative pathology affects these subregions differentially; neuropathological and neuroimaging studies indicate region-specific vulnerability and altered recruitment within prefrontal circuits across disease stages (Van Hoesen et al., 2000). Therefore, this study focuses on intra-prefrontal directional network organization as an intermediate-level analysis between single-region activation and whole-brain network topology.

Many connectivity studies in AD have relied on undirected functional connectivity, which quantifies statistical dependence but does not estimate the direction of information transfer. Effective connectivity approaches instead characterize directed interactions, estimating whether activity in one region provides predictive information about activity in another region (Friston, 1994; Valdes-Sosa et al., 2011). In dementia and prodromal impairment, directed connectivity analyses have reported altered interregional influence, suggesting that cognitive impairment may involve not only changes in connection strength but also changes in directionality (Afshari and Jalili, 2017; Renli et al., 2025). Information-theoretic measures such as transfer entropy (TE) provide a model-free approach for estimating directed information flow. TE has demonstrated sensitivity to disease-related changes in connectivity in EEG and fMRI studies of MCI and AD (Schreiber, 2000; McBride et al., 2015). However, most prior TE-based dementia studies have used EEG or fMRI, leaving unclear whether task-based functional near-infrared spectroscopy (fNIRS) can capture target-level directional convergence within prefrontal networks.

Although fMRI provides brain-wide coverage and higher spatial resolution, fNIRS is a practical modality for investigating task-related cortical network dynamics, particularly within superficial prefrontal regions, owing to its tolerance to movement, compatibility with cognitive tasks, and sensitivity to hemodynamic changes (Naseer and Hong, 2015; Dang et al., 2023). fNIRS studies have consistently reported altered prefrontal activation and functional connectivity in MCI and AD during executive and semantic tasks (Metzger et al., 2016; Yoo et al., 2020; Lee et al., 2024). However, studies examining directional effective connectivity using fNIRS remain limited (Bu et al., 2019), and many existing fNIRS studies instead focus on resting-state or undirected measures, limiting insight into task-dependent directional information flow within prefrontal circuits.

Task condition can alter directional prefrontal connectivity across clinically defined cognitive groups. Task-based paradigms such as semantic fluency, working memory, and inhibitory control engage distinct prefrontal subcircuits and can reveal altered prefrontal recruitment patterns that differ between MCI and AD (Herrmann et al., 2008; Yeung et al., 2016; Clément et al., 2013). Recent studies indicate that connectivity alterations in AD are often nonlinear, task-dependent, and vary across clinical stages and task conditions (Mijalkov et al., 2023; Zhang et al., 2025). These findings support the need for comparative analysis of directed prefrontal connectivity across multiple task conditions rather than exclusive reliance on resting-state data.

Estimating effective connectivity from empirical neuroimaging data requires rigorous statistical validation, as transfer entropy is sensitive to embedding parameters, delay selection, and finite-sample effects (Wibral et al., 2013; Bossomaier et al., 2016). Surrogate-based testing and stringent control of multiple comparisons are essential to reduce noise-driven estimates and improve the reliability of TE-based interaction estimates (Benjamini and Hochberg, 1995). Therefore, in addition to examining individual directed edges, this study summarized the mean incoming TE from multiple source regions to each prefrontal target. This target-level convergence approach was used to identify whether specific prefrontal regions received altered directional input across groups.

Within this framework, the present study examines task- and rest-related differences in directed effective connectivity within the prefrontal cortex across healthy control (HC), MCI, and AD groups using fNIRS and transfer entropy. Rather than emphasizing isolated connections, the analysis focuses on target-level convergence of directional information flow toward specific prefrontal subregions and on how these patterns differ across task conditions and clinically defined groups. This approach is intended to complement large-scale network studies by providing a subsystem-level perspective on prefrontal information integration across HC, MCI, and AD groups. Identifying such directional convergence patterns may help reveal stage-associated connectivity markers and provide insight into how information flow within executive prefrontal systems differs across HC, MCI, and AD groups.

Based on these objectives and prior evidence of prefrontal alterations in MCI and AD across task-based and resting-state conditions (Metzger et al., 2016; Yang and Hong, 2021), we hypothesized that directional effective connectivity would show target-level convergence patterns involving specific prefrontal targets rather than nonspecific edge-level alterations. Specifically, we expected that cross-sectional differences between MCI and AD would involve redistribution of directional information flow toward specific prefrontal targets, reflecting altered integration within prefrontal executive-control circuits. Given that resting state and cognitive tasks place distinct demands on prefrontal control circuits, we further hypothesized that these convergence patterns would differ across experimental conditions, with task conditions revealing condition-specific directional alterations beyond resting-state organization.

## 2 Materials and methods

### 2.1 Participants

In this study, 60 participants (HC: 16, MCI: 23, and AD: 21) were included. Healthy controls were recruited from the local community, whereas participants with MCI and AD were recruited from the rehabilitation and geriatric departments of Pusan National University Yangsan Hospital (Baik et al., 2022). Participants were recruited between October 2018 and September 2019. All participants were right-handed native Korean speakers aged 55–85 years with comparable educational backgrounds. Clinical diagnoses and group assignments were established by the hospital’s clinical team based on clinical evaluations and standardized cognitive assessments, including the Korean version of the Mini-Mental State Examination (K-MMSE) (Han et al., 2008), the Korean version of the Montreal Cognitive Assessment (K-MoCA) (Lee et al., 2008), and the Clinical Dementia Rating (CDR) (Morris, 1993). Individuals with a history of neurological or psychiatric disorders, including schizophrenia, were excluded.

No participant was excluded after recruitment; therefore, all 60 participants were included in the primary analysis. Task-specific missing data are described in the preprocessing section.

The study was approved by the institutional review board of Pusan National University Yangsan Hospital and conducted in accordance with the Declaration of Helsinki (World Medical Association, 2013). Comprehensive experimental details were explained to each participant, and a signed consent form was obtained before the experiment began. Table 1 summarizes the demographic information, cognitive scores, and statistical details for all participants.

**Table 1:** Demographic and clinical characteristics of participants across groups.

Characteristics	HC (*n* = 16)	MCI (*n* = 23)	AD (*n* = 21)	*p*-value
Sex (male: female)	7: 9	12: 11	10: 11	0.8716
Age (mean ± SD)	65.5 ± 5.67	74.13 ± 4.7	78.14 ± 6.26	<0.0001*
Education Years (mean ± SD)	11.12 ± 2.73	10.48 ± 2.78	9.29 ± 4.06	0.2971
K-MMSE score (mean ± SD)	28.50 ± 1.67	23 ± 2.39	15.05 ± 1.88	<0.0001*
K-MoCA (mean ± SD)	26.19 ± 2.01	17.39 ± 3.77	7.19 ± 2.60	<0.0001*
CDR (mean ± SD)	0.16 ± 0.24	0.48 ± 0.46	1.21 ± 0.68	<0.0001*
Hypertension [n (%)]	2/16 (12.5%)	6/23 (26.1%)	3/21 (14.3%)	0.362
Hyperlipidaemia [n (%)]	2/16 (12.5%)	5/23 (21.7%)	2/21 (9.5%)	0.965
Diabetes [n (%)]	2/16 (12.5%)	2/23 (8.7%)	4/21 (19.0%)	0.142

Values are presented as mean ± standard deviation (SD) for continuous variables and n (%) for categorical variables. Group differences were assessed using one-way ANOVA for continuous variables and chi-square tests for categorical variables. Cognitive scores (K-MMSE, K-MoCA, and CDR) were adjusted for age and years of education using ANCOVA. An asterisk (*) indicates statistically significant group differences (p < 0.05). HC: healthy controls; MCI: mild cognitive impairment; AD: Alzheimer’s disease; K-MMSE: Korean Mini-Mental State Examination; K-MoCA: Korean Montreal Cognitive Assessment; CDR: Clinical Dementia Rating. Bold values and an asterisk (*) indicates statistically significant group differences (*p* < 0.05).

### 2.2 Experiment design and task paradigm

**Figure 1:**
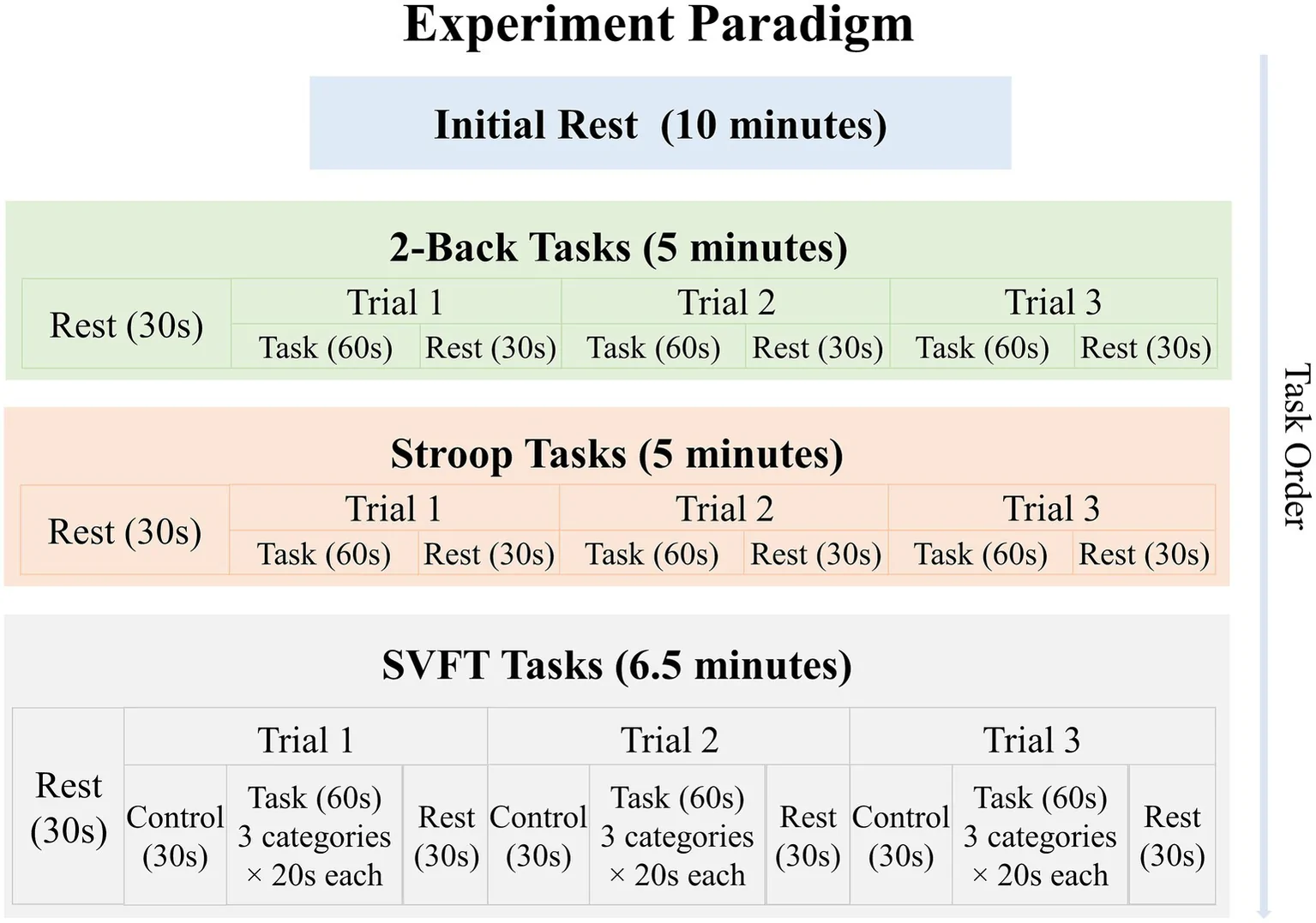
Experimental paradigm and task sequence.

The experimental paradigm shown in Figure 1 consisted of a ten-minute initial rest with eyes open, fixating on a screen, followed by three cognitive task sessions. The 2-back task was used to evaluate a subject’s working memory performance (Owen et al., 2005; Kane et al., 2007), and the Stroop task assessed inhibitory control, in which interference occurs when one stimulus attribute influences the processing of another (Scarpina and Tagini, 2017). The semantic verbal fluency task (SVFT) was also administered to assess vocabulary size, updating, lexical access speed, and inhibition ability in participants (Shao et al., 2014).

Participants were asked to sit comfortably in a chair and instructed to avoid movement as much as possible. The task order was fixed across participants (initial rest → 2-back → Stroop → SVFT). Each task session consisted of 3 trials, with each trial including a 60-s task period and a 30-s rest period. There was also a 30-s rest period separating consecutive task sessions. The initial 30-s rest shown at the beginning of each task block in Figure 1 served as the inter-session rest separating consecutive task sessions. First, resting-state data were acquired to minimize potential carryover effects of task-evoked cognitive engagement on baseline neural activity, consistent with prior fNIRS and neuroimaging protocols in aging and dementia research (Metzger et al., 2016; Yeung et al., 2016; Herrmann et al., 2008). Then the 2-back task was performed, in which a randomly selected number between 1 and 9 appeared on the screen. The participant was instructed to press the key on the keyboard when the current number matched the number displayed 2 trials earlier.

After the 2-back task, there was a 30-s rest, followed by the Stroop task. In this study, the Korean Color Word Stroop Test (K-CWST) was used in the Stroop task. Participants were required to report the ink color of the printed words (red, blue, green, or black) within a limited time period (Lee et al., 2021). Following the Stroop task, there was another 30-s rest before the SVFT began. In the SVFT task, participants were asked to generate as many words as possible from a specified semantic category (e.g., animals, fruits, or tools) within a limited time. Each task period in an SVFT trial was further divided into three 20-s word categories. A 30-s control period preceded each SVFT trial to allow participants to prepare for the task; however, this period was not included in the connectivity analysis. The SVFT assessed the efficiency of semantic memory retrieval under time constraints.

### 2.3 fNIRS data acquisition and probe configuration

**Figure 2:**
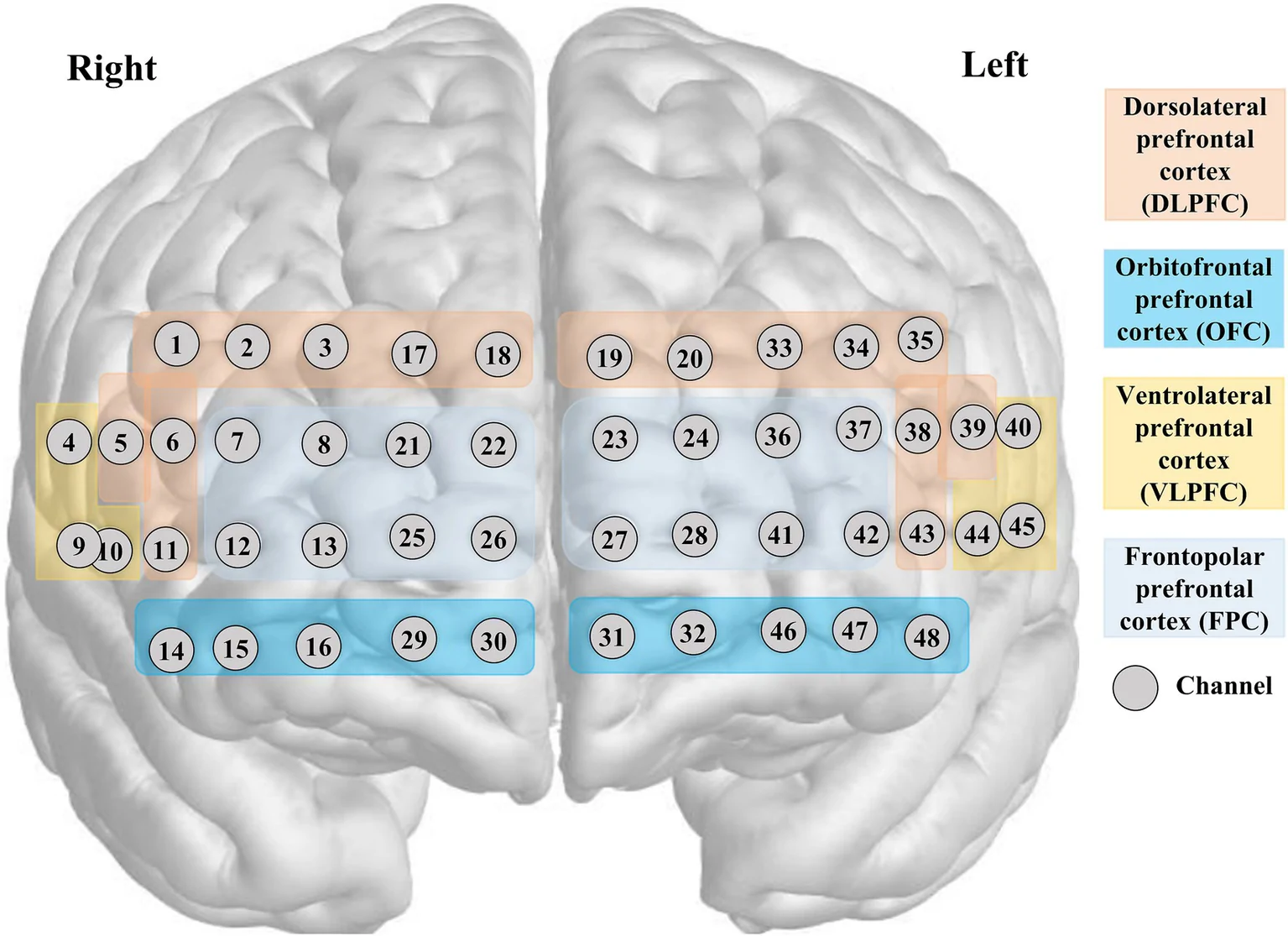
fNIRS channel layout and regional parcellation of the prefrontal cortex: Channel numbers indicate measurement locations used for regional connectivity analyses. The channel-layout illustration was adapted from OBELAB Inc. (2022), NIRSIT Channel Information. © 2022 OBELAB Inc.

In this study, data were acquired using NIRSIT (OBELAB Inc., Rep. of Korea), a multi-channel, continuous-wave (CW) fNIRS system (Yang et al., 2019) operating at a sampling rate of 8.138 Hz. To detect two chromophores [i.e., oxygenated hemoglobin (HbO) and deoxygenated hemoglobin (HbR)], two wavelengths of 780 nm and 850 nm were used. The system consists of 24 emitters and 32 detectors and is used to measure hemodynamic activity in the prefrontal cortex. In total, 48 channels with a source-detector distance of 30 mm, shown in Figure 2, were selected for analysis according to the EEG 10–20 system, with FpZ as the reference (Singh et al., 2005; Tsuzuki and Dan, 2014). Only channels with a standard 30-mm source-detector separation were included to maintain sensitivity to cortical hemodynamic signals. Although the montage included shorter 15-mm channels, these were not used for short-channel regression because they were not dedicated short-separation measurements optimized for superficial physiological regression and may include non-negligible cortical sensitivity.

### 2.4 Preprocessing

All preprocessing steps were performed in MATLAB (R2023b) using a customized pipeline. Raw optical intensity time-series data were first screened for nonphysical values. Nonphysical intensity samples (≤ 0) were considered as missing and handled using a two-stage strategy: short missing segments (≤ 2 s) were linearly interpolated, while any remaining missing samples were imputed using the channel-wise fifth percentile of valid (> 0) intensity values computed within each subject and task. The occurrence of nonphysical values was logged for quality control. Optical intensity signals were converted to optical density by computing the logarithmic change relative to the median intensity of the initial 5-s baseline period. Optical density changes were converted to HbO and HbR concentration changes using the modified Beer–Lambert law (MBLL) (Delpy et al., 1988; Sassaroli and Fantini, 2004). Differential pathlength factors were computed using an age- and wavelength-dependent model (Zhao et al., 2002) and incorporated into the MBLL conversion along with the 3-cm source-detector separation.

MBLL was followed by a linear detrending to remove slow baseline trends. Motion-related artifacts were then attenuated using a wavelet-kurtosis-based correction approach (kurtosis threshold: 2.5), which has been shown to effectively suppress abrupt motion-induced signals in fNIRS data (Chiarelli et al., 2015). The motion-corrected signals were filtered using a cascade filtering approach. First, a 4th-order Butterworth high-pass filter with a cutoff frequency of 0.01 Hz was applied to reduce slow baseline drifts. Subsequently, a 4th-order Butterworth low-pass filter with a cutoff frequency of 0.10 Hz was applied to attenuate higher-frequency physiological components, including respiratory and cardiac activity. Both filters were applied using zero-phase filtering to avoid phase distortion. This cutoff range was selected based on commonly used fNIRS preprocessing practice and to preserve low-frequency hemodynamic fluctuations while attenuating higher-frequency physiological noise; however, Mayer-wave activity near 0.1 Hz may be attenuated but not completely eliminated (Pinti et al., 2019).

As transfer entropy is sensitive to temporal dynamics, to evaluate pure task-related coupling, the task period from each trial was separated. Baseline correction was then applied to each trial task period by subtracting the mean of a 30-s pre-task rest (i.e., the rest period of the previous trial for the 2nd and 3rd trials, and the inter-task session rest used for the 1st trial). All task trials were subsequently z-scored within each subject and condition to facilitate comparison across channels.

Due to the lower spatial resolution of fNIRS compared to fMRI, channels were grouped into four prefrontal functional subregions: the dorsolateral prefrontal cortex (DLPFC), the ventrolateral prefrontal cortex (VLPFC), the frontopolar cortex (FPC), and the orbitofrontal cortex (OFC). Each region was represented separately in the left and right hemispheres, yielding eight regional nodes for connectivity analysis. Channel grouping was based on the manufacturer’s anatomical mapping (OBELAB Inc., 2022) and verified by projecting Montreal Neurological Institute (MNI) coordinates and cross-referencing them with Brodmann labels using the Talairach Daemon in NIRS-SPM (Ye et al., 2009; Lancaster et al., 2000). Regional definitions followed standard neuroanatomical criteria: DLPFC primarily encompassed Brodmann areas (BA) 9 and 46 with overlap into BA 8 and 10 (Nelson and Luciana, 2008; Mitchell, 2011; Kringelbach, 2005); VLPFC included BA 44, 45, and 47 with overlap into BA 11 and 12 (O'Reilly, 2010; Nelson and Luciana, 2008; Mitchell, 2011); OFC comprised BA 11 and 47 with contributions from BA 10, 12, and 13 (BioSource Software, 2025; Kringelbach, 2005); and FPC was defined as regions with 90% overlap within BA 10 (BioSource Software, 2025; Peng et al., 2018). Figure 2 illustrates the frontal probe montage and channel layout.

Principal component analysis (PCA) was applied separately for each trial, region, hemoglobin signal, task, and subject. Principal components cumulatively explaining at least 90% of the variance were retained. Each retained component was reconstructed into a time-series signal, and when multiple components were retained, the reconstructed signals were averaged to produce a single representative signal for the region, thereby reducing noise and channel-specific variability. Signal variance, power spectral density, and signal-to-noise ratio were monitored at each preprocessing stage to ensure data integrity. Three subjects were missing the 2-back task session (1 HC and 2 AD), and 1 HC subject was missing the Stroop session due to an incomplete task session. These subjects were included in the analysis for the available task and resting-state sessions.

**Figure 3:**
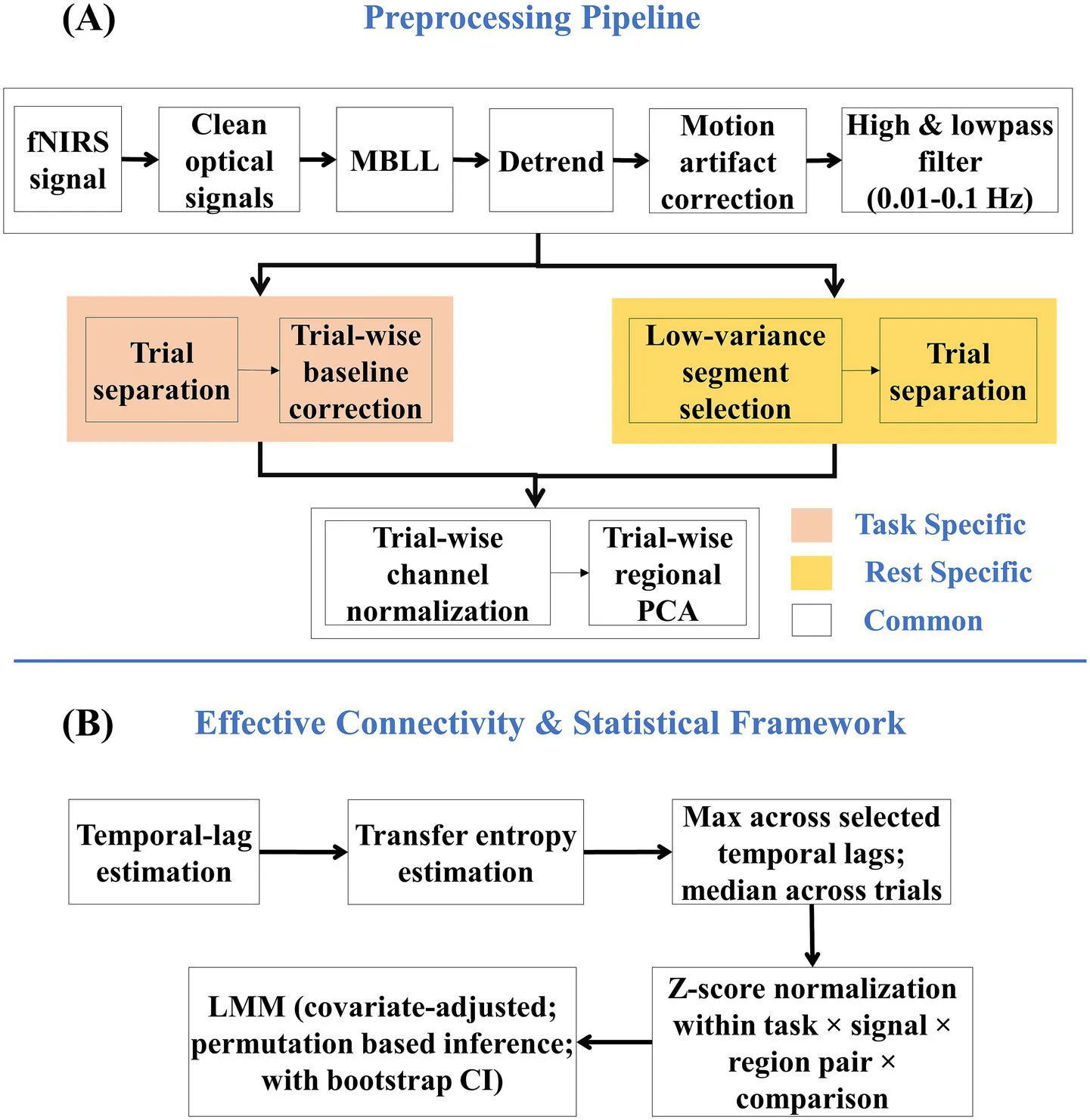
Preprocessing pipeline and effective connectivity analysis framework **(A)** fNIRS preprocessing included signal cleaning, conversion using the modified Beer–Lambert law (MBLL), detrending, motion correction, and bandpass filtering (0.01–0.1 Hz). Task data were segmented into trials with baseline correction, while resting-state data underwent low-variance segment selection. All data were normalized and reduced to region-wise signals using PCA. **(B)** Effective connectivity was estimated using transfer entropy (TE) after temporal lag selection. TE values were summarized across lags and trials, z-score-normalized within each condition, and analyzed using covariate-adjusted linear mixed-effects models (LMM) with permutation-based inference.

To make the resting-state data comparable with the task sessions, a 5-min resting-state segment with minimal signal variance across channels was first identified and then divided into three disjoint 60-s trials. Baseline correction was not applied to resting-state data; however, all other preprocessing steps were applied to the resting-state data in the same way as to task-related data. The preprocessing pipeline for resting-state and tasks is summarized in Figure 3.

### 2.5 Effective connectivity estimation using transfer entropy

Directed effective connectivity (EC) was estimated between prefrontal subregions using transfer entropy (TE), introduced by Schreiber (2000), which is an information-theoretic measure of directed statistical dependence from a source time series *X* to a target time series *Y*, conditional on the target’s own past. Unlike mutual information, TE is time-lagged and directional, making it suitable for estimating directed information flow in continuous and nonlinear neurophysiological signals (Schreiber, 2000; Wibral et al., 2013; Bossomaier et al., 2016). Formally, TE from *X → Y* is the conditional mutual information between the past of *X* and the future of *Y*, conditioned on the past of *Y*, as shown in Equation 1:

$$TE_{X\to Y}^{\left(m,l\right)}=I(Y_{t+1};X_{t}^{\left(l\right)}|Y_{t}^{\left(m\right)})$$ (1)

Equation 1 is equivalent to the difference between two conditional entropies, as shown in Equation 2:

$$TE_{X\to Y}^{\left(m,l\right)}=H(Y_{t+1}|Y_{t}^{\left(m\right)})-H(Y_{t+1}|Y_{t}^{\left(m\right)},X_{t}^{\left(l\right)})$$ (2)

The embedded source and target histories are defined in Equations 3 and 4, respectively:

$$X_{t}^{\left(l\right)}=\{X_{t-(l-1)\tau_{x}},\ldots ,X_{t-\tau_{x}},X_{t}\}$$ (3)

$$Y_{t}^{\left(m\right)}=\{Y_{t-(m-1)\tau_{y}},\ldots ,Y_{t-\tau_{y}},Y_{t}\}$$ (4)

Where *l* and *m* are the source and target history lengths, respectively, and *τ_x_* and *τ_y_* are the corresponding embedding delays. To account for source–target interaction delays, we used the lagged form of TE shown in Equation 5, consistent with prior work on information-transfer delay estimation (Wibral et al., 2013).

$$TE_{X\to Y}^{\left(m,l,u\right)}=I(Y_{t+1};X_{t+1-u}^{\left(l\right)}|Y_{t}^{\left(m\right)})$$ (5)

Where *u* > 0 denotes the candidate source–target interaction delay.

Pairwise TE was calculated between all predefined prefrontal subregions, excluding self-connections, yielding 56 directed connections per trial, per hemoglobin signal (HbO, HbR), and per experimental condition (rest, 2-back, Stroop, SVFT).

An adaptive lag approach was employed to accommodate region-specific hemodynamic delays. For each source–target region pair within each trial, signal, task, and subject, mutual information (MI) was computed over a range of delays (1 to 75 samples; approximately 0.12–9.2 s). The three delays with the highest MI values were selected as candidate source–target delays. TE was then estimated separately for each delay using the Kraskov–Stögbauer–Grassberger (KSG) k-nearest-neighbor estimator (*k* = 5) (Kraskov et al., 2004), and the embedding parameters (history length and history delay) were optimized using the auto-embedding method (Ragwitz criterion) (Ragwitz and Kantz, 2002). For each source–target pair within each trial, this procedure produced three delay-specific TE estimates corresponding to the three MI-selected candidate delays.

To obtain a single trial-level TE estimate for each source-target pair, the maximum TE value among the three selected candidate delays was retained. No sliding-window TE or local transfer entropy time series was analyzed; for each candidate delay, TE was estimated at the trial level as a single average TE value for each source-target pair. This lag-selection strategy was applied uniformly across all subjects and groups to accommodate variability in hemodynamic response timing rather than to optimize group differences. A subject-level TE estimate for each directed connection was obtained by taking the median across trials. Median aggregation was chosen to reduce sensitivity to trial-specific variability and outliers. Thus, for each subject, task, and hemoglobin signal, the final TE matrix contained 56 directed connection values. The Java Information Dynamics Toolkit (JIDT) was used to compute TE estimates (Lizier, 2014), accessed via Python.

### 2.6 Statistical analysis

Group differences in demographic variables were assessed using one-way analysis of variance (ANOVA) for continuous measures (age and education years), followed by Tukey’s honestly significant difference (HSD) tests for pairwise comparisons. Categorical variables (sex distribution and vascular risk factors including hypertension, hyperlipidemia, and diabetes) were compared using Pearson’s chi-square tests.

Group differences in cognitive and clinical scores (K-MMSE, K-MoCA, and CDR) were evaluated using analysis of covariance (ANCOVA), with age included as a covariate. *Post hoc* pairwise comparisons were conducted using estimated marginal means with Holm correction to control for multiple comparisons. Statistical significance was set at *p* < 0.05.

The effective-connectivity primary analysis was conducted at the subject level, using TE values standardized within Task × Signal × directed region-pair restricted to the two groups included in each pairwise comparison prior to modeling. This within-connection standardization ensured comparability across edges while preserving between-group differences and allowing fixed-effect estimates to be interpreted as standardized effects expressed in standard deviation units.

To evaluate group differences, linear mixed-effects models (LMMs) were fitted for each task, hemoglobin signal, and directed region-pair connection across pairwise group comparisons (e.g., MCI vs. AD, HC vs. MCI, and HC vs. AD). For each subject, task, and hemoglobin signal, the effective-connectivity data consisted of an 8 × 8 directed TE matrix across the eight predefined prefrontal regions. After excluding self-connections, each matrix contained 56 directed region-pair TE values. Statistical testing was performed separately for each directed region pair, not on all 56 values simultaneously. Thus, for each Task × Signal × Comparison × directed region-pair model, the dependent variable was a single subject-level TE value for each participant included in the corresponding pairwise contrast. In each LMM, the dependent variable was the z-scored TE value. Group was included as a fixed effect, with the first group in each comparison serving as the reference group. Age (mean-centered across all subjects) and sex (binary coded) were used as covariates, with subject ID included as a random intercept. Models were estimated using maximum likelihood (REML = False) with the L-BFGS optimizer as implemented in the *statsmodels* Python library.

Because of a small sample size and potential heteroscedasticity, asymptotic Wald *p*-values may be unreliable, particularly in mixed-effects models with clustered data; hence, statistical inference was based on Monte Carlo empirical *p*-values using the Freedman-Lane residual permutation test (Freedman and Lane, 1983; Winkler et al., 2014). In this procedure, a reduced model excluding the group term was first fitted, and the residuals from this reduced model were permuted at the subject level. The permuted residuals were added to the reduced model-fitted values to generate permuted outcomes, and the full model, including group and covariates, was re-estimated. This procedure was repeated 3,000 times per connection, and at least 100 valid permutation replicates were required for inference. Two-sided empirical *p*-values were computed using the absolute value of the Group coefficient with a + 1 correction. This approach preserves the dependence structure induced by the covariates in the design matrix and provides valid nonparametric inference for the group effect even in the presence of potential deviations from normality.

To quantify uncertainty, 95% confidence intervals (CIs) were estimated using a stratified subject-level bootstrap procedure. Subjects were resampled with replacement within each group, preserving clustering by resampling entire subject-level blocks. For each of 3,000 bootstrap replicates, the mixed-effects model was re-estimated using the same z-scored outcome variable. Importantly, the original standardization was retained and not recomputed within bootstrap samples, ensuring consistent effect scaling across replicates. Percentile-based 95% confidence intervals were derived from the bootstrap distribution of beta estimates, requiring a minimum of 100 valid bootstrap replicates for interval estimation.

Multiple comparisons were controlled using the Benjamini–Hochberg false discovery rate (BH-FDR) procedure (Benjamini and Hochberg, 1995). Correction was applied at the target level within families, defined by Task × Signal × Comparison, across edges converging on the same target region. This target-wise correction strategy was based on prespecified *a priori* hypotheses that disease-related alterations would manifest as directional changes in information flow converging on specific prefrontal target regions. Each target-wise FDR family included the seven incoming directed edges converging on a given target region.

### 2.7 Sensitivity analysis

Additional sensitivity analyses were conducted to assess the robustness of the primary findings by examining estimator parameterization, covariate specification, and surrogate-based normalization.

#### 2.7.1 Sensitivity to KSG k-NN parameter

TE estimation stability with respect to the nearest-neighbor parameter in the KSG estimator was evaluated by recomputing TE with *k* = 4 (primary analysis: *k* = 5). Embedding dimensions and source-target delay parameters selected in the primary analysis were held fixed to isolate the effect of varying the nearest-neighbor parameter. The full linear mixed-effects modeling and permutation-based inference pipeline was re-applied for the significant primary findings. This analysis was restricted to connections significant in the primary model. Robustness was evaluated based on the consistency of the effect direction, CIs not crossing zero, and comparable effect magnitude relative to the primary model, defined as a change in the standardized effect size of ≤ 30% from the primary estimate.

#### 2.7.2 Covariate sensitivity

To assess the effects of covariate adjustment, models for the significant findings were re-estimated, excluding age and sex separately. A finding was considered robust to covariate specification if (i) the direction of the effect remained unchanged, (ii) the 95% CI did not cross zero, (iii) the empirical permutation-based *p*-value remained below 0.1, and (iv) the standardized effect size differed by no more than 30% relative to the primary model. The 30% threshold was used as a heuristic stability criterion to detect substantial attenuation of the effect. If the effect size decreased by more than 30% while all other parameters remained the same, the findings were considered attenuated; if the 95% CI crossed zero or the permutation-based p-value exceeded 0.1, the findings were considered fragile to covariate specification.

#### 2.7.3 Surrogate-based validation

To further evaluate whether observed group differences reflected structured directional interactions beyond autocorrelation-driven effects, surrogate-based normalization was performed as a complementary validation step. This analysis was restricted to target families identified in the primary target-wise analysis, consistent with our a priori hypothesis of target-level convergence.

For each directed connection in the significant target families, phase-randomized surrogate time series were generated (Prichard and Theiler, 1994) for the source signals (*X*) while preserving the target signal (*Y*). Embedding dimensions and delay parameters were frozen at the values selected in the primary analysis. For each trial, the surrogate TE was computed using the delay set from the primary analysis, and a max-statistic approach was applied, consistent with the delay-selection strategy. Trial-level surrogate TE values were then aggregated using the median across trials. This procedure was repeated 5,000 times for each subject-connection.

For each subject, an empirical surrogate-normalized Z statistic was computed as the standardized deviation of the observed max-statistic median TE value relative to its surrogate distribution. Subject-level *Z* values were then averaged across connections converging on the same target region for a specific task and signal, yielding a target-level surrogate-normalized connectivity measure per subject. To ensure consistency across group comparisons, the set of inbound connections used for averaging was prespecified and fixed for each Task × Signal × Target combination. Target-level averaging was performed over the same set of directed edges that were significant in the primary analysis for that specific Task × Signal × Comparison × Target family. The same edge set was used to compute both the primary family-level summary and the surrogate-normalized summary, ensuring direct comparability of effect magnitudes. This approach ensured that surrogate validation evaluated the robustness of the same hypothesis-defined convergence pattern within each comparison. The same linear mixed-effects modeling and Monte Carlo permutation-based inference framework described in the primary analysis was applied to these target-level surrogate-normalized outcomes.

Surrogate-based robustness was interpreted as follows:

- **S**table finding: effect direction consistent with the primary model, bootstrap confidence interval not crossing zero, and empirical permutation *p*-value < 0.10.
- Attenuated but supported finding: effect direction consistent, confidence interval not crossing zero, empirical permutation *p*-value < 0.10, but >30% attenuation in magnitude relative to the primary family-level effect.
- Partially stable finding: effect direction consistent but with either confidence interval crossing zero or empirical *p*-value ≥ 0.10, indicating attenuation of statistical strength while preserving directional tendency.
- Unstable finding: reversal of effect direction or collapse of statistical support under surrogate normalization.
- Contradictory finding: reversal of effect direction accompanied by empirical *p*-value < 0.10, suggesting sensitivity of inference to surrogate normalization.

Surrogate validation was not used to redefine statistical significance but to assess the robustness of directional group differences under null-model normalization.

### 2.8 Exploratory ROC analysis

To explore the potential translational relevance of the identified target-level convergence patterns, receiver operating characteristic (ROC) analyses were conducted using repeated stratified cross-validation (5 folds × 50 repeats). For each binary contrast, features were defined as the mean incoming transfer entropy (TE) across significant inbound edges within the corresponding Task × Signal × Target families identified in the primary LMM-based analysis. Because feature definition was based on connections identified in the full dataset, this ROC analysis should be interpreted as an exploratory *post hoc* assessment of potential discriminative utility. The statistically identified convergence families differed across MCI vs. AD, HC vs. AD, and HC vs. MCI comparisons; therefore, the ROC feature sets were contrast-specific and could involve different Task × Signal × Target combinations. Accordingly, AUC values were interpreted within each binary contrast and were not used to rank the intrinsic diagnostic separability of HC, MCI, and AD groups.

To maintain consistency with the covariate-adjusted inferential framework of the study and to prevent information leakage, feature residualization with respect to age (mean-centered) and sex was performed within each training fold only. Specifically, linear regression models were fitted on the training set to remove age and sex effects from each feature, and residualized values were then applied to both training and held-out test data within that fold prior to classification.

A logistic regression classifier (balanced class weights) was trained on residualized features. Model performance was evaluated using out-of-fold (OOF) predicted probabilities aggregated across repeats. The primary performance metric was the area under the ROC curve (AUC). Confidence intervals for OOF AUC were estimated using stratified bootstrap resampling at the subject level (2,000 iterations), preserving class proportions. Thus, ROC findings were interpreted as exploratory evidence of potential discriminative utility, not as optimized diagnostic classification performance.

## 3 Results

For the primary effective-connectivity analysis, each subject contributed one 8 × 8 directed TE matrix for each task and hemoglobin signal, corresponding to the eight predefined prefrontal regions. Self-connections were excluded, yielding 56 directed region-pair TE values per subject for each Task × Signal condition. Statistical testing was performed separately for each directed region pair; therefore, each Task × Signal × Comparison × directed region-pair model included one subject-level TE value per participant in the corresponding pairwise contrast. Before task-specific missing-session handling, the pairwise comparison sample sizes were 44 for MCI vs. AD, 39 for HC vs. MCI, and 37 for HC vs. AD. Because some task sessions were incomplete, task-specific analyses used the available sessions as described in Section 2.4.

### 3.1 Demographic and clinical characteristics

Demographic and clinical characteristics are summarized in Table 1. Age differed significantly across groups (one-way ANOVA, *p* < 0.0001). Tukey *post hoc* tests indicated that both MCI and AD participants were significantly older than healthy controls (both *p* < 0.001), whereas the age difference between MCI and AD did not reach statistical significance. After adjusting for age, significant group effects were observed for K-MMSE, K-MoCA, and CDR (all ANCOVA *p* < 0.0001). Holm-adjusted post hoc comparisons showed lower K-MMSE and K-MoCA scores in MCI and AD compared with HC, with the lowest scores observed in AD (all adjusted *p* < 0.05). For CDR, the AD group exhibited significantly higher scores than both HC and MCI (both adjusted *p* < 0.001), whereas the HCMCI contrast did not reach statistical significance.

No significant differences were observed in sex distribution (*p* = 0.8716), years of education (*p* = 0.2971), or vascular risk factors, including hypertension, hyperlipidemia, and diabetes (all *p* > 0.05). These findings indicate that, aside from age and disease-related cognitive measures, the groups were broadly comparable in demographic composition and vascular comorbidity burden. Given the significant age differences across groups, subsequent connectivity analyses used covariate-adjusted modeling frameworks to account for potential confounding effects of age and sex.

### 3.2 Target-level convergence in the MCI vs. AD comparison

Across all tasks and hemoglobin signals, the MCI vs. AD comparison revealed 55 directed connections surviving target-wise FDR correction. The resulting target-level convergence patterns and their task–signal distributions are summarized in Table 2. Of these, 53 connections showed bootstrap confidence intervals that did not cross zero, consistent with stable directional effects under permutation-based inference.

**Table 2:** Summary of target-specific alterations in directional effective connectivity across tasks and signal types in MCI vs. AD.

Target	Task	Signal	Direction	No. of sources	β-range	q-range	CI robustness
FPC-R	2-Back	HbR	Decreased	7	[−0.76, −0.89]	[0.002, 0.006]	7/7 robust
FPC-R	SVFT	HbR	Decreased	7	[−0.72, −0.81]	[0.008, 0.012]	7/7 robust
DLPFC-L	2-Back	HbO	Increased	7	[0.61, 0.84]	[0.017,0.025]	7/7 robust
DLPFC-L	SVFT	HbO	Increased	7	[0.66, 0.82]	[0.010,0.011]	7/7 robust
DLPFC-L	Stroop	HbR	Increased	6	[0.56, 0.85]	[0.030,0.045]	6/6 robust
FPC-L	Stroop	HbR	Increased	7	[0.67, 0.85]	[0.007,0.017]	7/7 robust
OFC-L	Stroop	HbO	Increased	7	[0.57, 0.76]	[0.039,0.042]	7/7 robust
VLPFC-L	Stroop	HbO	Decreased	7	[−0.53, −0.72]	[0.038,0.047]	5/7 robust

This table summarizes significant target-level patterns of directed effective connectivity derived from transfer entropy (TE) analysis. For each target region, results are aggregated across all significant source-to-target connections identified using linear mixed-effects models (LMMs), with multiple-comparison correction. “Direction” indicates whether connectivity is increased or decreased in AD relative to MCI. “No. of Sources” denotes the number of significant source regions contributing to the target. β-range represents the range of standardized LMM effect sizes across contributing edges, and q-range indicates the corresponding range of FDR-corrected p-values. “CI Robustness” reflects the proportion of connections for which bootstrap confidence intervals did not include zero. Abbreviations: DLPFC, dorsolateral prefrontal cortex; FPC, frontopolar cortex; OFC, orbitofrontal cortex; VLPFC, ventrolateral prefrontal cortex; HbO, oxygenated hemoglobin; HbR, deoxygenated hemoglobin; SVFT, semantic verbal fluency task.

These significant connections converged on five unique prefrontal target regions, supporting a non-uniform distribution of disease-related alterations. Among these targets, DLPFC-L and FPC-R emerged as the most prominent target regions for convergent directional alterations, each receiving multiple directed connections across tasks. The remaining targets exhibited fewer or task-specific convergent effects. Task-wise distribution indicated that the Stroop paradigm accounted for the largest proportion of significant directed alterations in the MCI vs. AD comparison, suggesting that executive-control demands were associated with more detectable directional connectivity differences between MCI and AD. Signal-wise distribution was broadly comparable between HbR (*n* = 27) and HbO (*n* = 26) among robust findings, indicating that disease-related effects were not confined to a single hemodynamic measure. However, subsequent sensitivity analyses revealed greater stability of HbR-based effects under surrogate normalization and parameter variation (see Section 3.8).

**Figure 4:**
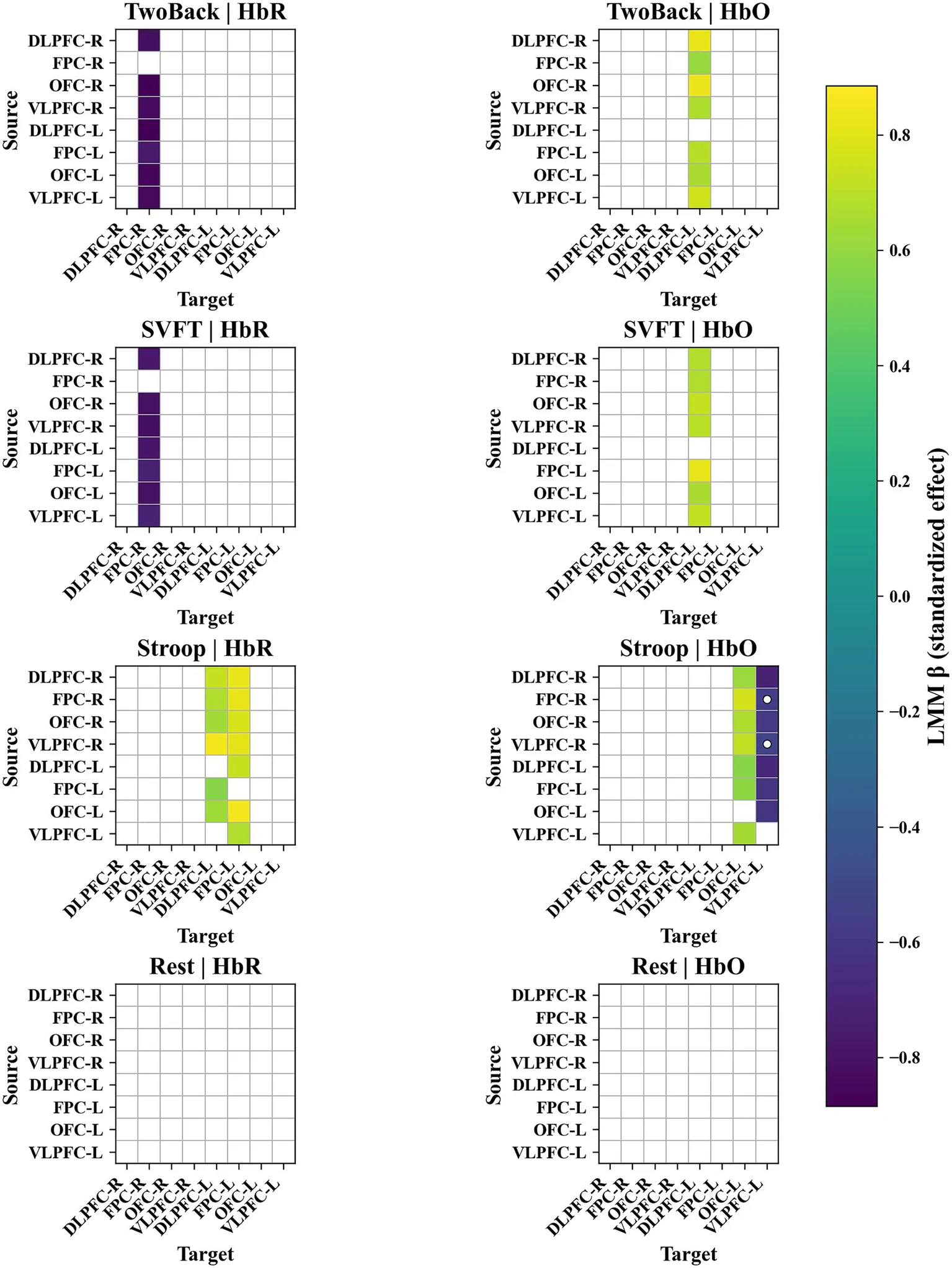
Task- and signal-specific target-level alterations in directed information flow (MCI vs. AD). Heatmaps display standardized linear mixed-effects model (LMM) β estimates of transfer entropy (TE) for each source-target region pair across tasks (TwoBack, Stroop, SVFT, Rest) and signal types (HbO, HbR). Positive values indicate increased connectivity in AD relative to MCI, while negative values indicate reduced connectivity. All region pairs are shown for completeness; colored cells indicate connections surviving multiple-comparison correction, while empty cells represent non-significant effects. White dots denote region pairs where the bootstrap confidence interval includes zero, indicating reduced stability of the estimated effect. Rows represent source regions and columns represent target regions.

These findings supported the primary hypothesis that cross-sectional differences between MCI and AD were characterized not by diffuse connectivity changes, but by preferential convergence of directional alterations on specific prefrontal targets, particularly DLPFC-L and FPC-R (Figure 4). Subsequent sections describe the principal convergence patterns underlying these target-level disease-related alterations.

### 3.3 Convergent reduction of information flow to FPC-R in MCI vs. AD

Right frontopolar cortex (FPC-R) emerged as a prominent convergence target in the MCI vs. AD comparison. Across both the 2-back and SVFT tasks, multiple directed connections from prefrontal source regions converged onto FPC-R, consistently showing reduced information transfer in AD compared with MCI.

In the 2-back task (HbR), seven source-to-FPC-R connections survived target-wise FDR correction. All effects were negative, consistent with reduced directed influence toward FPC-R in AD. Standardized effect sizes ranged from −0.756 to −0.885, with corresponding FDR-corrected *q*-values between 0.002 and 0.006 (Supplementary Table 1). Bootstrap confidence intervals for all connections did not cross zero, confirming the stability of these effects under resampling.

A similar pattern was observed in the SVFT task (HbR), where directed connections from all other prefrontal sources to FPC-R were again significantly reduced in AD relative to MCI. Effect sizes ranged from −0.715 to −0.813 (Supplementary Table 1), with *q*-values between 0.0083 and 0.0117. All associated confidence intervals excluded zero.

**Figure 5:**
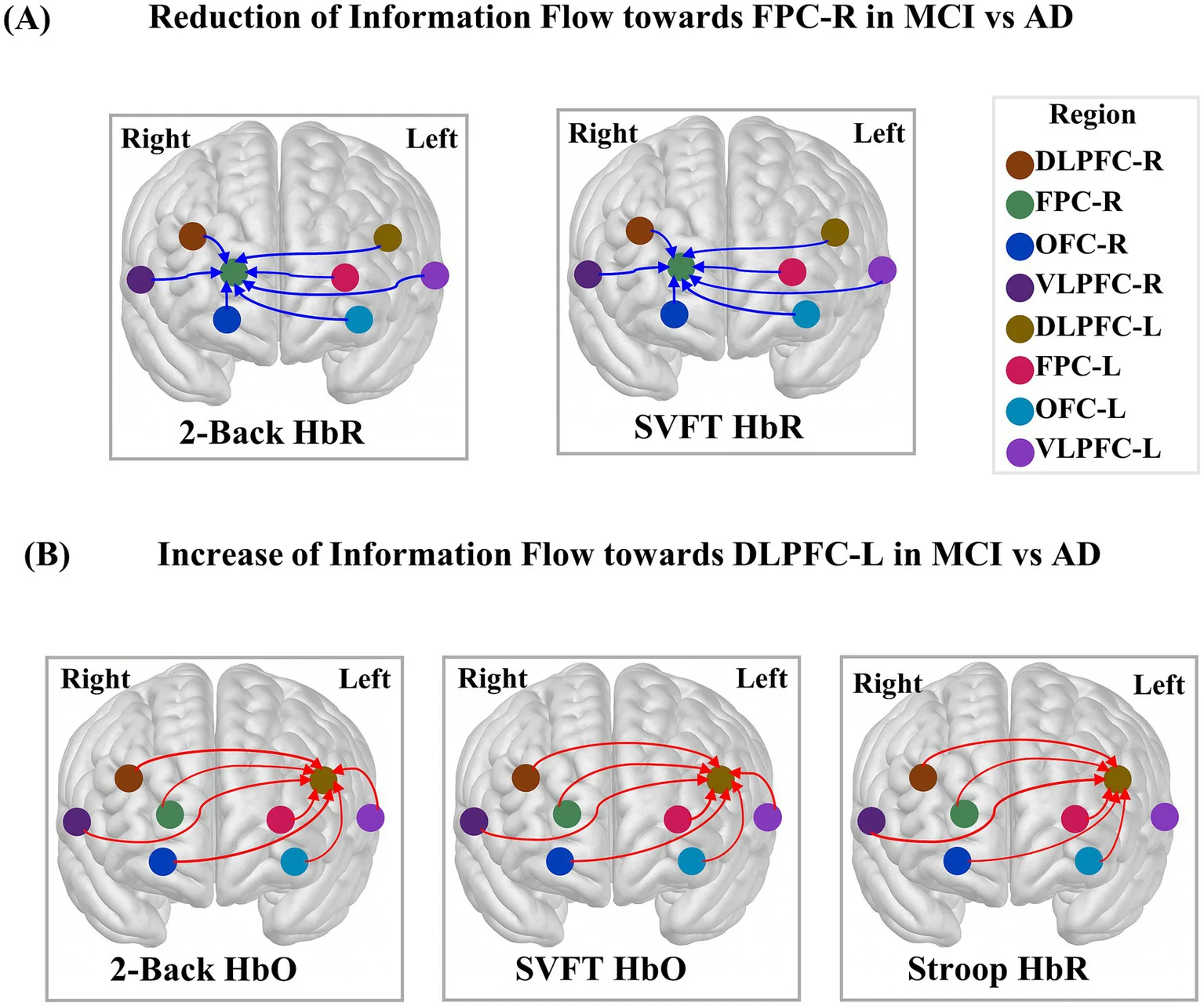
Redistribution of directional information flow in AD relative to MCI. **(A)** Reduced information flow toward the right frontopolar cortex (FPC-R) in AD compared to MCI, observed in HbR signals during the 2-back and SVFT tasks. **(B)** Increased information flow toward the left dorsolateral prefrontal cortex (DLPFC-L) in AD compared to MCI, observed in HbO (2-back and SVFT) and HbR (Stroop) conditions. Arrows indicate the direction of information flow between regions, with red representing increased connectivity and blue representing decreased connectivity. Arrow thickness is proportional to the magnitude of the standardized LMM *β* values. The brain-background illustration was adapted from OBELAB Inc., (2022), NIRSIT Channel Information, and cleaned using OpenAI ChatGPT 4.5. OBELAB’s prescribed citation instructions were followed. All regional nodes, arrows, labels, colors, and connectivity results were created and verified by the authors. © 2022 OBELAB Inc.

Importantly, the directional consistency across two cognitively distinct paradigms, namely working memory (2-back) and semantic fluency (SVFT), indicated that reduced convergence onto FPC-R was not limited to a single task but consistent with a repeated disease-related alteration in prefrontal information flow (Figure 5A). These results supported the hypothesis that FPC-R was identified as a prominent convergence target in AD relative to MCI, characterized by widespread attenuation of directional inputs across executive and semantic task contexts.

### 3.4 Convergent increase of directed influence toward DLPFC-L in MCI vs. AD

In contrast to the reduction observed for FPC-R, the left dorsolateral prefrontal cortex (DLPFC-L) showed consistent convergence of increased directional influence in AD relative to MCI. Directed connections from distributed prefrontal source regions converged onto DLPFC-L across all three task paradigms.

In the 2-back and SVFT tasks (HbO), all seven possible source regions exhibited significant increases in directed information transfer toward DLPFC-L in AD. For the 2-back task, standardized effect sizes ranged from 0.613 to 0.835 (Supplementary Table 1), with FDR-corrected *q*-values ranging from 0.0169 to 0.0253. In the SVFT task, effect sizes ranged from 0.658 to 0.818, and *q*-values ranged from 0.0105 to 0.0113. All associated bootstrap confidence intervals excluded zero, consistent with robust directional effects.

A similar pattern was observed during the Stroop task (HbR), in which six source regions showed significantly greater directional influence toward DLPFC-L in AD than in MCI. Effect sizes ranged from 0.564 to 0.854 (Supplementary Table 1), with *q*-values between 0.0303 and 0.0447. Again, none of the confidence intervals crossed zero.

Notably, this convergence pattern differed from that observed for FPC-R in both directionality and signal distribution. Whereas FPC-R showed consistent reductions in HbR-based directional input, DLPFC-L exhibited increases in directed influence predominantly in HbO (2-back and SVFT) and HbR (Stroop). The presence of convergent effects across three distinct cognitive paradigms indicated that DLPFC-L emerged as a prominent convergence target in AD relative to MCI, characterized by increased incoming directional interactions (Figure 5B).

Together, the opposing patterns observed for FPC-R (reduced convergence) and DLPFC-L (increased convergence) suggested a redistribution of prefrontal directional information flow in AD relative to MCI, rather than uniform global disruption.

### 3.5 Summary of convergence patterns in MCI vs. AD

Taken together, the MCI vs. AD findings revealed a structured redistribution of directional information flow across prefrontal targets. While FPC-R showed widespread attenuation of incoming influence across working memory and semantic fluency tasks (HbR), DLPFC-L exhibited consistent augmentation of convergent inputs across all three paradigms, spanning both HbO and HbR signals. Importantly, these effects were not driven by isolated connections but rather reflected multi-source convergence onto specific targets, thereby supporting a non-random reorganization pattern in the MCI vs. AD comparison. The opposing directionality, reduced convergence toward FPC-R while increasing convergence toward DLPFC-L, was consistent with a differential redistribution of directional inputs across prefrontal targets (Figure 5). These opposing convergence patterns across tasks and signals are summarized in Table 2.

### 3.6 Stroop-specific convergent alterations in MCI vs. AD

Beyond the cross-paradigm effects observed in FPC-R and DLPFC-L, the Stroop task revealed additional target-level convergence patterns specific to executive-control demands.

In Stroop HbR, the left frontopolar cortex (FPC-L) showed significant convergence from all seven prefrontal source regions. Directed information flow toward FPC-L was increased in AD relative to MCI, with standardized effect sizes ranging from 0.673 to 0.852 and FDR-corrected *q*-values between 0.007 and 0.017 (Supplementary Table 1). All associated bootstrap confidence intervals excluded zero, consistent with stable task-specific augmentation of directional input.

Similarly, in Stroop HbO, the left orbitofrontal cortex (OFC-L) showed greater directional influence from all seven source regions in AD than in MCI. Effect sizes ranged from 0.573 to 0.764, with q-values between 0.0387 and 0.0423 (Supplementary Table 1). Confidence intervals did not cross zero for these connections.

In contrast, Stroop HbO revealed convergent reductions toward the left ventrolateral prefrontal cortex (VLPFC-L), where AD exhibited decreased directed information flow relative to MCI. Effect sizes ranged from −0.532 to −0.718, with *q*-values between 0.0382 and 0.047 (Supplementary Table 1). However, two of the seven directed connections toward VLPFC-L had bootstrap confidence intervals that crossed zero, consistent with partial instability of this pattern relative to other Stroop-specific targets.

Collectively, these findings suggested that the Stroop paradigm was associated with additional target-level differences in AD relative to MCI, including increased convergence onto FPC-L and OFC-L and decreased convergence onto VLPFC-L. However, unlike FPC-R and DLPFC-L, these effects appeared task-dependent rather than cross-paradigm (Figure 4).

### 3.7 HC vs. AD and HC vs. MCI comparisons

In contrast to the extensive convergence observed in the MCI vs. AD comparison, the HC-based contrasts showed fewer, more task-restricted target-level alterations.

#### 3.7.1 HC vs. AD

Across all tasks and signals, 12 directed connections survived FDR correction in the HC vs. AD comparison, of which 9 had confidence intervals that excluded zero. Detailed edge-level statistics for these contrasts are provided in Supplementary Table 1.

The most consistent convergence pattern emerged during the Stroop task (HbO), where six prefrontal source regions showed increased directed influence toward OFC-L in AD relative to HC. Standardized effect sizes ranged from 0.833 to 1.084, with *q*-values between 0.0438 and 0.0447. All associated confidence intervals excluded zero, consistent with stable executive task-specific augmentation of directional input toward OFC-L in AD.

Additionally, during the 2-back task (HbR), directed connections from six source regions converged onto FPC-R, showing reduced information transfer in AD compared to HC (effect size range: −0.87 to −1.18; *q* = 0.0396). However, three of these six connections exhibited confidence intervals crossing zero, consistent with partial instability of this pattern relative to the corresponding MCI vs. AD findings.

Overall, HC vs. AD effects were fewer in number and more restricted to individual tasks compared to the broader convergence observed in the MCI vs. AD comparison (Supplementary Figures 1, 3A).

#### 3.7.2 HC vs. MCI

In the HC vs. MCI comparison, 14 directed connections survived FDR correction.

During resting-state HbR, all seven prefrontal source regions converged onto DLPFC-L, demonstrating increased directed information flow in MCI relative to HC. Standardized effect sizes ranged from 0.899 to 1.012, with *q*-values between 0.0101 and 0.0103. All associated confidence intervals excluded zero, consistent with robust MCI-associated augmentation of convergent influence toward DLPFC-L.

In the 2-back task (HbO), all seven source regions converged onto VLPFC-R, showing decreased information transfer in MCI compared with HC (effect sizes ranging from −0.776 to −1.088; *q*-values ranging from 0.0289 to 0.032). None of these connections showed confidence intervals that crossed zero.

These findings suggested that HC vs. MCI alterations were characterized by selective convergence onto DLPFC-L at rest and reduced task-driven input toward VLPFC-R, whereas MCI vs. AD comparison showed broader redistribution patterns affecting FPC-R and DLPFC-L across multiple paradigms (Supplementary Figures 2, 3B). Complete statistics for individual directed edges are reported in Supplementary Table 1.

### 3.8 Sensitivity analyses

#### 3.8.1 Sensitivity to KSG parameter (*k* = 4)

Re-estimation of TE using *k* = 4 yielded patterns highly consistent with the primary analysis (*k* = 5), with convergence robustness summarized in Supplementary Table 2.

In the MCI vs. AD comparison, convergent reductions toward FPC-R during 2-back and SVFT remained robust, with preserved effect direction, non-crossing confidence intervals, and comparable standardized effect sizes (≤ 30% deviation). Similarly, convergent increases toward DLPFC-L across 2-back, SVFT, and Stroop tasks remained stable under *k* = 4 re-estimation.

Among Stroop-specific findings, HbR FPC-L effects remained robust under *k* = 4. For Stroop HbO OFC-L, effect direction and magnitude were preserved, and empirical permutation *p*-values remained < 0.10; however, three connections exhibited a confidence interval crossing zero, consistent with mild attenuation relative to the primary model. In contrast, Stroop HbO VLPFC-L showed instability under *k* = 4, with confidence intervals crossing zero for all primary significant edges.

For HC vs. MCI, resting-state HbR DLPFC-L and 2-back HbO VLPFC-R remained robust under *k* = 4. In HC vs. AD, 2-back HbR FPC-R effects preserved direction and magnitude but exhibited confidence intervals crossing zero for all but one edge. Stroop HbO OFC-L remained largely stable, with only one connection showing a confidence interval crossing zero.

Overall, the principal convergence patterns identified in MCI vs. AD, particularly those involving FPC-R and DLPFC-L, were robust to variation in the KSG nearest-neighbor parameterization (Supplementary Table 2).

#### 3.8.2 Covariate sensitivity

Sensitivity results for models excluding age as a covariate are summarized in Supplementary Table 3. Re-estimation of models excluding sex did not materially alter any significant findings, with all primary effects retaining direction, comparable magnitude, and non-crossing confidence intervals (Supplementary Table 4).

Sensitivity to age adjustment revealed selective attenuation patterns. In the MCI vs. AD comparison, convergent reductions toward FPC-R during 2-back and SVFT remained robust after excluding age as a covariate, with preserved direction, confidence intervals excluding zero, and empirical permutation *p*-values < 0.10. Similarly, DLPFC-L convergence during 2-back and SVFT remained robust. For Stroop HbR DLPFC-L, all but one connection retained robustness; one edge became fragile due to the confidence interval crossing zero.

Among Stroop-specific MCI vs. AD findings, HbR FPC-L effects were largely stable, with one connection classified as fragile when age was excluded as a covariate. For Stroop HbO OFC-L, four edges retained robustness, whereas three became fragile. Stroop HbO VLPFC-L showed greater age sensitivity, with all but two edges classified as fragile.

In HC vs. AD, all significant edges became fragile after excluding age as a covariate, indicating that HC vs. AD contrast findings were sensitive to age adjustment. In HC vs. MCI, resting-state HbR DLPFC-L effects were attenuated, and 2-back HbO VLPFC-R findings were largely classified as fragile, with only one edge classified as attenuated rather than fragile.

Overall, the principal MCI vs. AD convergence patterns, particularly those involving FPC-R and DLPFC-L during 2-back and SVFT, remained robust to covariate specification, whereas Stroop-specific and HC-based contrasts exhibited greater sensitivity to exclusion of age as a covariate (Supplementary Table 3).

#### 3.8.3 Surrogate-based validation

Surrogate-based normalization was performed to assess whether the observed group differences reflected directional interactions that exceeded phase-randomized null expectations. The stability classification across targets and tasks is summarized in Supplementary Table 5.

In the MCI vs. AD comparison, the 2-back HbR convergence toward FPC-R remained stable under surrogate normalization, preserving effect direction, non-crossing confidence intervals, and empirical permutation *p*-values < 0.10. However, SVFT HbR FPC-R effects became unstable following surrogate normalization, with reversal of effect direction relative to the primary model, confidence intervals crossing zero, and empirical permutation *p*-values > 0.10.

For DLPFC-L, 2-back and SVFT effects were partially stable under surrogate normalization, with preserved direction but attenuation of statistical strength relative to the primary model. In contrast, Stroop HbR DLPFC-L effects became unstable under surrogate normalization, showing reversal of effect direction accompanied by confidence intervals crossing zero and empirical *p*-values > 0.10.

Among Stroop-specific findings in MCI vs. AD, HbR FPC-L, HbO OFC-L, and HbO VLPFC-L patterns were classified as partially stable, retaining consistent directional trends but exhibiting reduced statistical robustness under null-model normalization.

In HC vs. AD, both 2-back HbR FPC-R and Stroop HbO OFC-L were attenuated under surrogate validation, retaining directional consistency but showing >30% change in effect magnitude relative to the primary family-level effects. In HC vs. MCI, resting-state HbR DLPFC-L and 2-back HbO VLPFC-R were partially stable under surrogate normalization, preserving effect direction but showing reduced statistical support (confidence intervals crossing zero and empirical permutation *p*-values ≥ 0.10).

Overall, surrogate-based validation supported stability of the strongest MCI vs. AD effect (2-back HbR FPC-R), showed attenuation in HC vs. AD contrasts, indicated partial stability in HC vs. MCI target families and in the remaining MCI vs. AD target families, and identified selective instability characterized by direction reversal in SVFT HbR FPC-R and Stroop HbR DLPFC-L effects. Importantly, surrogate normalization was used as a complementary robustness assessment and did not redefine statistical significance (Supplementary Table 5).

### 3.9 Exploratory ROC analysis

To evaluate the potential discriminative utility of the identified target-level convergence patterns, exploratory ROC analyses were performed using repeated stratified cross-validation (5 folds × 50 repeats) with covariate-adjusted residualization of features within each training fold. Each ROC analysis therefore tested whether the convergence features identified for the corresponding contrast carried discriminative information within that same binary contrast.

**Figure 6:**
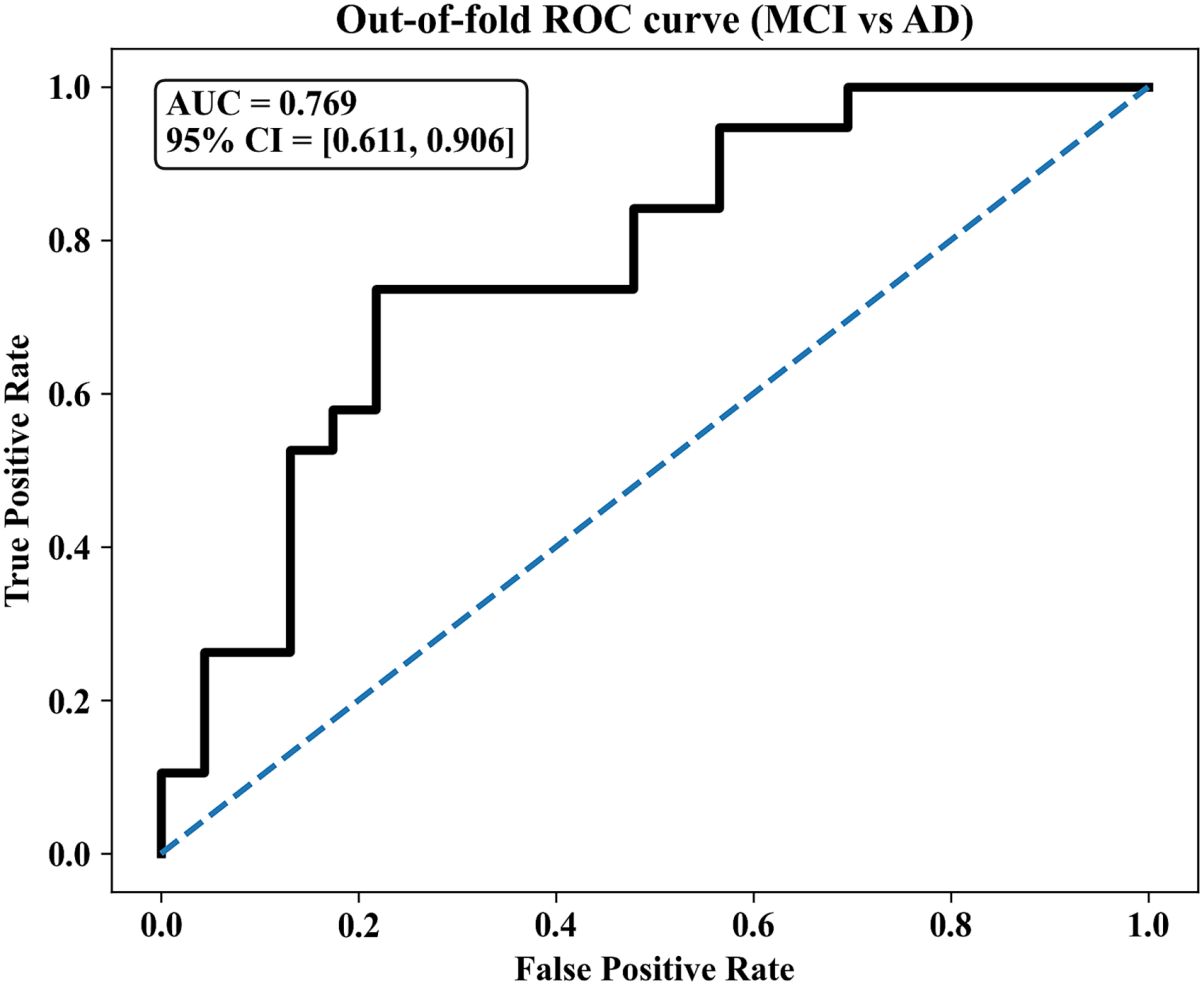
Exploratory ROC analysis of the classification of MCI and AD using target-level connectivity features. The dashed diagonal line represents chance-level performance. True positive rate (sensitivity) is plotted against false positive rate (1-specificity).

For the MCI vs. AD contrast, two features were included: mean convergence toward DLPFC-L (2-back HbO) and mean convergence toward FPC-R (2-back HbR). Repeated cross-validation yielded a mean fold AUC of 0.778 (*SD* = 0.164; 250 folds). The aggregated out-of-fold (OOF) AUC was 0.769. Subject-level stratified bootstrap estimation (2,000 iterations) produced a 95% confidence interval of [0.611, 0.906], indicating moderate discriminative utility for the selected TE-derived features, with uncertainty consistent with the sample size (Figure 6).

For the HC vs. AD comparison, features included mean convergence toward OFC-L (Stroop HbO) and FPC-R (2-back HbR). The covariate-adjusted repeated cross-validation yielded a mean fold AUC of 0.619 (*SD* = 0.241; 250 folds). The OOF AUC was 0.617. Subject-level bootstrap estimation yielded a 95% confidence interval of [0.417, 0.812], reflecting limited discriminative utility for the selected features with wide uncertainty (Supplementary Figure 4A).

For the HC vs. MCI comparison, features included mean convergence toward DLPFC-L (rest HbR) and VLPFC-R (2-back HbO). Repeated cross-validation yielded a mean fold AUC of 0.738 (*SD* = 0.223; 250 folds). The OOF AUC was 0.704. Subject-level bootstrap estimation yielded a 95% confidence interval of [0.527, 0.870], indicating modest discriminative utility with substantial uncertainty (Supplementary Figure 4B).

Among the contrast-specific exploratory ROC analyses, the selected TE-derived convergence features showed moderate discriminative utility for the MCI vs. AD comparison. This finding aligned with the target-level convergence patterns identified in the primary connectivity analyses for this contrast. Because different feature sets were used across contrasts, the AUC values were interpreted within each binary comparison and were not used to conclude that one clinical contrast was inherently easier to classify than another. However, the wide bootstrap confidence intervals across all contrasts reflected the limited sample size and the exploratory nature of the classification framework (Figure 6 and Supplementary Figure 4). These ROC analyses were therefore interpreted as exploratory assessments of potential translational relevance rather than as optimized diagnostic classification models.

## 4 Discussion

This study investigated directional effective connectivity within prefrontal cortical networks across clinically defined HC, MCI, and AD groups using transfer entropy (TE) applied to fNIRS signals recorded during rest and three cognitive paradigms. The primary objective was to assess whether clinical group differences were associated with structured differences in directional information flow rather than diffuse alterations in connectivity strength. The MCI vs. AD comparison showed target-specific convergence patterns of directional connectivity. Specifically, reduced incoming information flow to the right frontopolar cortex (FPC-R) was consistently observed during both the working memory (2-back) and semantic fluency (SVFT) tasks, whereas increased directional influence to the left dorsolateral prefrontal cortex (DLPFC-L) emerged across several task conditions. These results suggested that AD, relative to MCI, involved a redistribution of directed interactions within prefrontal networks, rather than uniform disruption of connectivity across regions. These conclusions were supported by multiple robustness analyses, including parameter sensitivity testing, covariate adjustments, and surrogate-based validation. Surrogate normalization supported the strongest MCI vs. AD effect (2-back HbR convergence toward FPC-R), whereas several task-specific patterns showed reduced stability under null-model normalization.

A consistent finding was a convergent reduction in the directional flow of information toward FPC-R across both working memory and semantic fluency paradigms. Across these tasks, multiple prefrontal source regions exhibited reduced directed influence toward FPC-R in AD compared with MCI, consistent with the idea that FPC-R received less integrated input in AD relative to MCI. The frontopolar cortex is recognized as a higher-order integrative region involved in coordinating complex cognitive processes, such as strategic planning, goal maintenance, and integration of multiple cognitive operations (Hogeveen et al., 2022). Reduced incoming information flow to this region may therefore reflect impaired coordination of distributed prefrontal signals necessary for demanding cognitive tasks. Reduced convergence toward FPC-R was evident across both paradigms in the primary (non-surrogate) analysis; however, the SVFT-related FPC-R pattern showed reduced stability under surrogate normalization, suggesting that cross-paradigm generalization should be interpreted with caution.

The observation of this pattern across two distinct paradigms suggests that the reduction in frontopolar convergence is not limited to a single cognitive domain but may reflect a broader alteration in prefrontal network organization in AD relative to MCI. However, this interpretation warrants caution, as TE derived from hemodynamic signals reflects directional statistical dependencies rather than direct neuronal causality (Vicente et al., 2011). Despite this limitation, the consistency of the pattern across paradigms and validation analyses supports the interpretation that reduced frontopolar integration may represent an important feature of directional network disruption in the MCI vs. AD comparison.

In contrast to the reduced convergence observed for FPC-R, the analysis identified increased directional influence toward DLPFC-L across several task conditions. This finding suggests that, in AD relative to MCI, more prefrontal regions directed information toward the dorsolateral prefrontal cortex. The DLPFC is central to executive functions, including working memory maintenance, attentional control, and monitoring task performance (Friedman and Robbins, 2022). Increased directional convergence toward this region may therefore reflect changes in the allocation of information flow within prefrontal networks during cognitive processing.

This pattern may reflect a network-level redistribution of directional influence, rather than simple strengthening of connectivity. Given the reduced FPC-R convergence observed in AD relative to MCI, increased DLPFC-L convergence may indicate redistribution of directional information flow within the prefrontal network. Similar patterns of increased prefrontal recruitment have been reported in studies of cognitive impairment and are often interpreted as adaptive or compensatory responses to declining network efficiency (Yoon et al., 2019). However, since the present analysis focuses on directional connectivity rather than neural activation, the observed increase should be interpreted as reorganization of information flow rather than definitive evidence of compensatory neural activity, although it is consistent with compensation hypotheses reported in prior task-based MCI studies. Collectively, the opposing patterns observed for FPC-R and DLPFC-L suggested that AD, relative to MCI, was characterized by reduced convergence toward FPC-R and increased convergence toward DLPFC-L, consistent with a redistribution of directional influence within prefrontal executive-control regions.

Another notable observation was the strong task dependence of alterations in directional connectivity. The most robust convergence patterns emerged in the working memory (2-back) and semantic fluency (SVFT) paradigms, whereas resting-state connectivity showed fewer stable alterations. These findings suggest that cognitively demanding tasks may reveal disease-related directional connectivity differences that are less evident during resting-state conditions.

Task paradigms engage specific neural systems that support goal-directed cognitive processing (Nau et al., 2024). The 2-back task imposes substantial demands on working memory and executive monitoring, both of which strongly recruit dorsolateral and frontopolar prefrontal regions (Owen et al., 2005; Ma et al., 2024). Similarly, semantic verbal fluency requires coordinated retrieval and executive-control processes supported by distributed prefrontal networks (Whitney et al., 2012). Under these task conditions, disease-related differences in directional information flow may become more evident than during rest. Additional convergence patterns observed during the Stroop task suggested that inhibitory-control demands may reveal task-specific directional connectivity alterations that were not observed in the 2-back, SVFT, or resting-state analyses. However, these effects showed reduced stability in sensitivity analyses and surrogate normalization; therefore, they should be interpreted with caution.

A major strength of this study is the systematic evaluation of the robustness of detected connectivity patterns. Sensitivity analyses showed that the principal convergence patterns, particularly those involving FPC-R and DLPFC-L, remained stable when alternative nearest-neighbor parameters were used in the TE estimation procedure. Similarly, excluding sex as a covariate did not materially alter the detected connectivity effects.

Age-related sensitivity analyses indicated that some findings, particularly those involving HC-based contrasts, were influenced by age adjustment. This outcome aligns with the significant age differences across participant groups and underscores the importance of controlling for demographic variables when interpreting connectivity differences. Surrogate-based normalization further showed that the strongest MCI vs. AD effect (2-back HbR convergence toward FPC-R) remained stable relative to phase-randomized null expectations, while several task-specific findings showed reduced stability. These results emphasize that not all detected edges necessarily reflect directional interactions exceeding surrogate-based null models, reinforcing the need for cautious interpretation of weaker or task-specific effects.

Exploratory ROC analyses were conducted to assess whether the identified convergence features could provide discriminative information between clinical groups. The selected TE-derived convergence features showed moderate discriminative utility for the MCI vs. AD comparison, particularly for target-level measures derived from 2-back connectivity patterns. Because these features were derived from convergence families identified in the primary inferential analysis, the ROC results should be interpreted as exploratory and hypothesis-driven rather than as an independently validated diagnostic model. By comparison, the selected features for HC-based contrasts showed more limited discriminative utility and wider uncertainty; however, because different feature sets were used across contrasts, these AUC values should not be interpreted as ranking the inherent classification difficulty of the clinical comparisons. This interpretation is consistent with prior reports that AD-related connectivity alterations can be heterogeneous and task-dependent rather than uniformly severity-proportional (Badhwar et al., 2017; Penalba-Sánchez et al., 2023). These analyses therefore offer only preliminary evidence that directional connectivity features may possess potential discriminative value and should be interpreted as exploratory rather than definitive.

Several limitations should be considered when interpreting these findings. The sample size was relatively modest, which may have limited statistical power and restricted generalizability. The fNIRS montage was limited to superficial prefrontal cortical regions, preventing assessment of broader cortical and subcortical networks implicated in dementia-related functional changes. TE estimates derived from hemodynamic signals reflect directional statistical dependencies rather than direct neuronal interactions, and vascular dynamics may influence the measured signals. Short-channel regression was not performed because the available 15-mm channels were not treated as dedicated short-separation regressors optimized for superficial physiological regression. In addition, direct monitoring of systemic physiology was not incorporated; therefore, residual extracerebral and systemic contributions cannot be fully excluded (Scholkmann et al., 2022; von Lühmann et al., 2020). Finally, the cross-sectional design does not allow direct observation of longitudinal changes in network connectivity within individuals. Since the task order was fixed (rest → 2-back → Stroop → SVFT), fatigue or order effects cannot be fully excluded. In addition, although age was included as a covariate in the statistical models, residual confounding related to group age differences may persist.

Future studies could extend these findings by examining directional changes in connectivity in larger cohorts. To enhance cortical coverage, researchers might incorporate multimodal neuroimaging approaches. For example, combining fNIRS with modalities such as EEG or fMRI could clarify how directional influences across spatially distributed networks are affected during cognitive decline. Future fNIRS studies may also incorporate dedicated short-separation channels and systemic physiology–augmented preprocessing pipelines to better isolate cortical components of directional connectivity. By following individuals longitudinally across stages of cognitive impairment, analyses could determine whether target-level convergence patterns serve as early indicators of disease progression. Integration of molecular biomarkers, such as amyloid or tau measures, could further clarify whether directional convergence patterns track underlying pathological burden. Ultimately, these approaches may support the development of noninvasive biomarkers to monitor functional network alterations associated with neurodegenerative disorders.

## 5 Conclusion

In summary, this study indicates that AD, relative to MCI, is associated with structured reorganization of directional information flow within prefrontal networks. Rather than showing uniform connectivity disruption, the MCI vs. AD comparison was characterized by reduced convergence of information flow toward the right frontopolar cortex and increased directional influence toward the left dorsolateral prefrontal cortex. Additional task-dependent alterations were observed under the demands of executive-control tasks. The strongest and most robust MCI vs. AD difference was observed in the working memory (2-back) condition. These findings highlight the importance of examining target-level directional network interactions to better understand functional reorganization across clinically defined cognitive groups.

## Data Availability

The datasets presented in this article are not readily available because data sharing is restricted due to participant privacy considerations and institutional review board regulations. Requests to access the datasets should be directed to Keum-Shik Hong. kshong@pusan.ac.kr.
