# Supplementary material for: Target-level convergence of directional prefrontal connectivity across Alzheimer’s disease stages: an fNIRS study
Source: Front Aging Neurosci. 2026 Aug 24;18:1834253. doi: 10.3389/fnagi.2026.1834253 (PMC13547515; doi:10.3389/fnagi.2026.1834253)
Supplement: Supplementary file 1 [file Data_Sheet_1.PDF]

Supplementary Material

1 Supplementary Figures and Tables

Following sections contain supplementary figures and tables.

1.1 Supplementary Figures

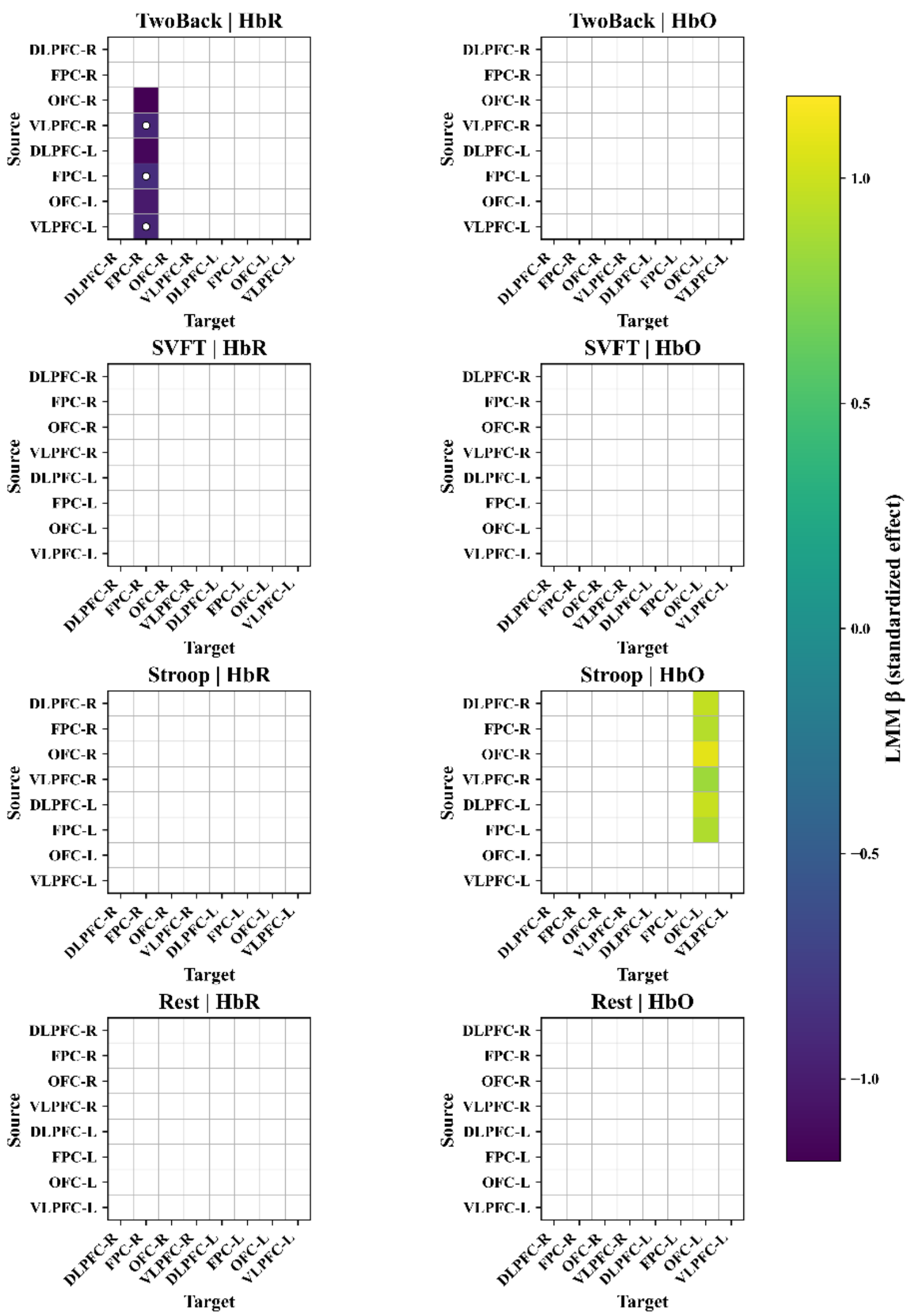

Supplementary Figure 1. Task-specific group differences in effective connectivity between HC and AD. Heatmaps display standardized linear mixed-effects model (LMM) β estimates of transfer entropy (TE) for each source–target region pair across tasks (TwoBack, Stroop, SVFT, Rest) and signal types (HbO, HbR). Positive values indicate increased connectivity in AD relative to HC, while negative values indicate reduced connectivity. All region pairs are shown for completeness; colored

cells indicate connections surviving multiple comparison correction, while empty cells represent non-significant effects. White dots denote region pairs where the bootstrap confidence interval includes zero, indicating reduced stability of the estimated effect. Rows represent source regions and columns represent target regions.

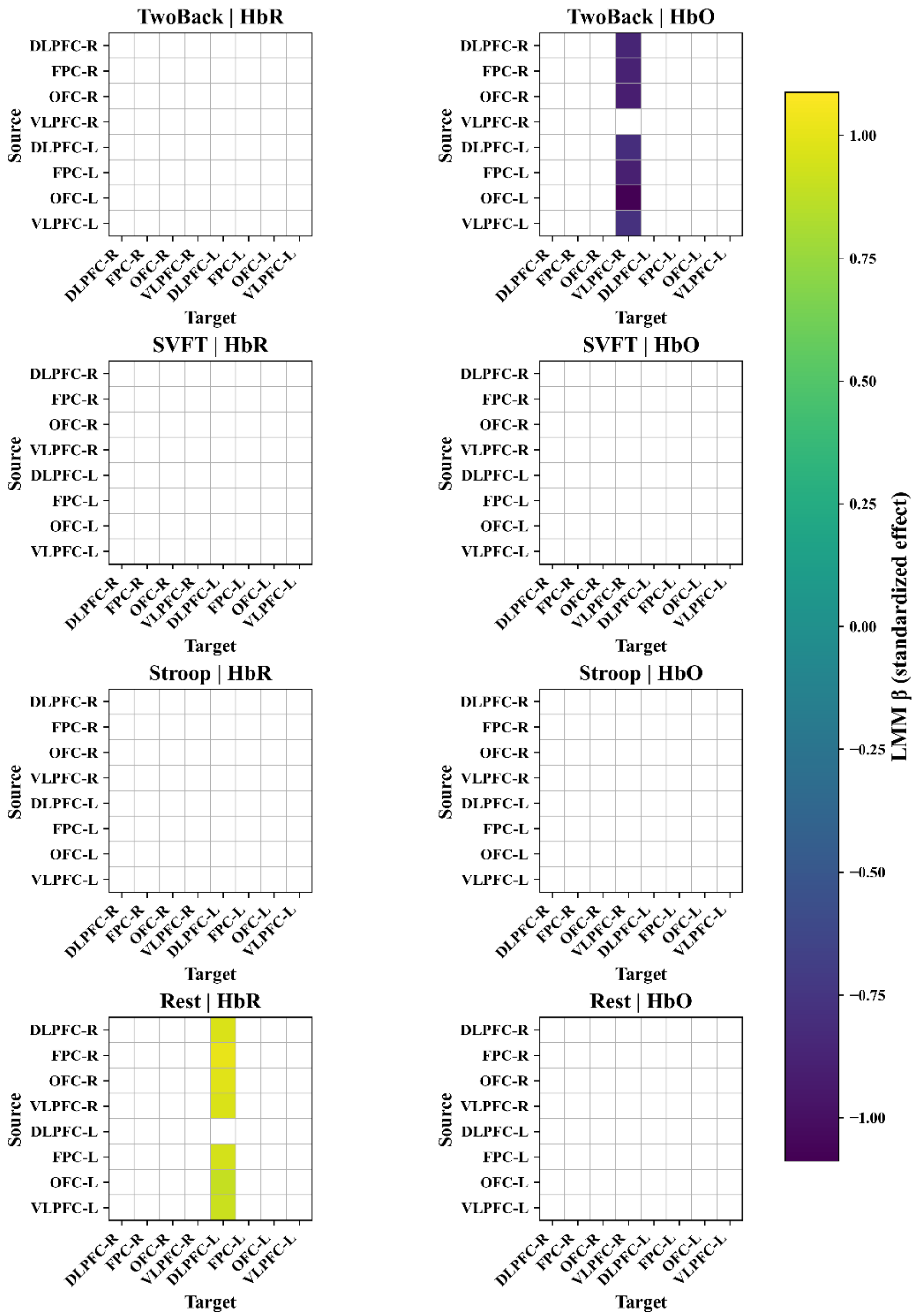

Supplementary Figure 2. Task-specific group differences in effective connectivity between HC and MCI. Heatmaps display standardized linear mixed-effects model (LMM) β estimates of transfer entropy (TE) for each source–target region pair across tasks (TwoBack, Stroop, SVFT, Rest) and signal types (HbO, HbR). Positive values indicate increased connectivity in MCI relative to HC, while negative values indicate reduced connectivity. All region pairs are shown for completeness;

colored cells indicate connections surviving multiple comparison correction, while empty cells represent non-significant effects. White dots denote region pairs where the bootstrap confidence interval includes zero, indicating reduced stability of the estimated effect. Rows represent source regions and columns represent target regions.

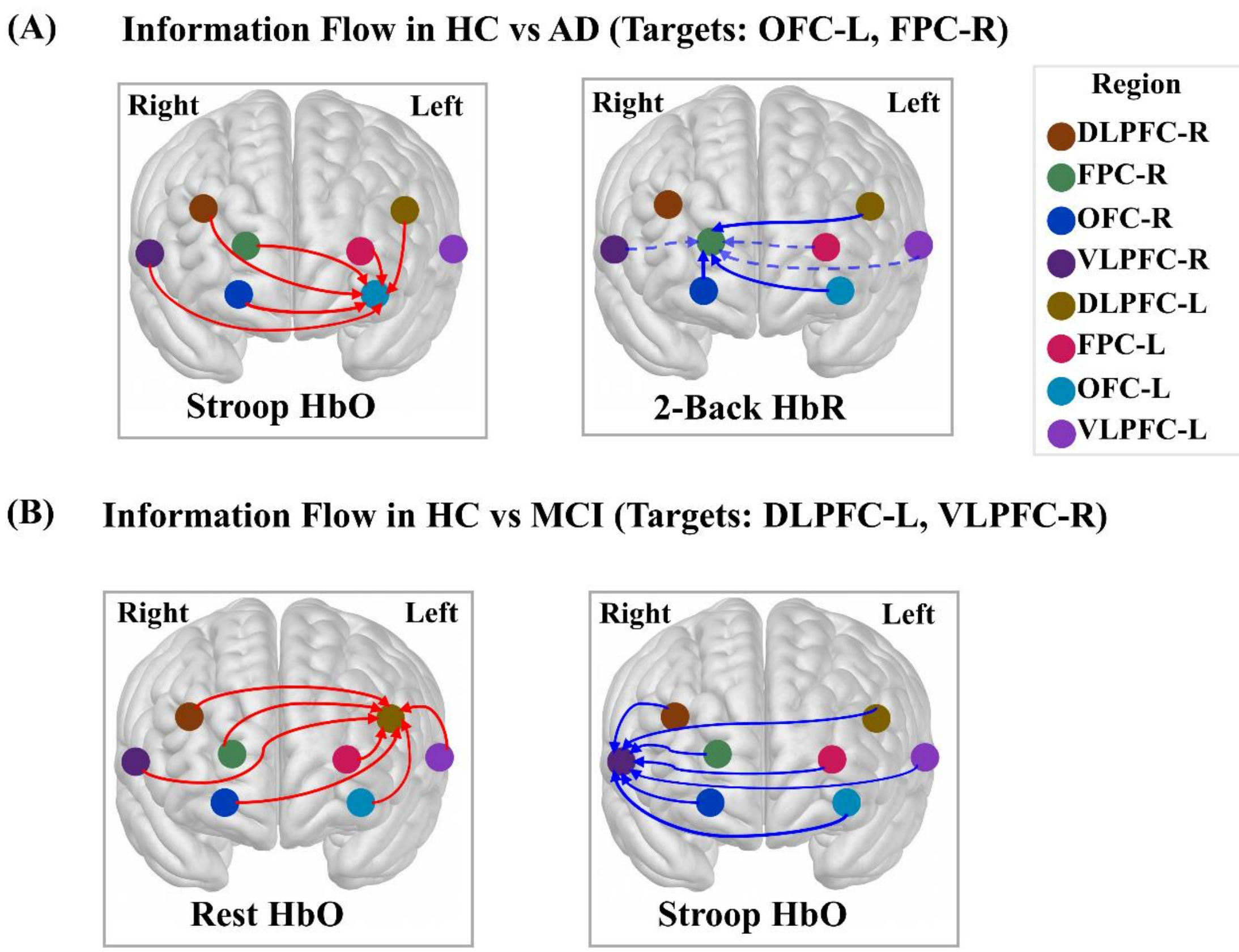

Supplementary Figure 3. Task-specific alterations in directional effective connectivity for HC vs. AD and HC vs. MCI. (A) HC vs. AD: Reduced (blue) and increased (red) information flow toward OFC-L and FPC-R during Stroop (HbO) and 2-back (HbR) tasks. (B) HC vs. MCI: Altered information flow toward DLPFC-L and VLPFC-R during Rest (HbO) and Stroop (HbO). Arrows represent significant directed connections identified using transfer entropy; red indicates increased connectivity and blue indicates decreased connectivity relative to the comparison group. Arrow thickness reflects the magnitude of the standardized LMM β estimates. Dotted arrows indicate connections for which the bootstrap confidence interval includes zero, reflecting reduced stability of the estimated effect. Nodes correspond to prefrontal regions as indicated in the legend, with left and right hemispheres labeled for orientation. The brain-background illustration was adapted from OBELAB, Inc. (2022), *NIRSIT Channel Information*, Seoul, Korea (https://www.obelab.com/info/notice.php), using OpenAI's ChatGPT 4.5 to produce a cleaned version. All regional nodes, arrows, labels, colors, and connectivity results were created and verified by the authors.

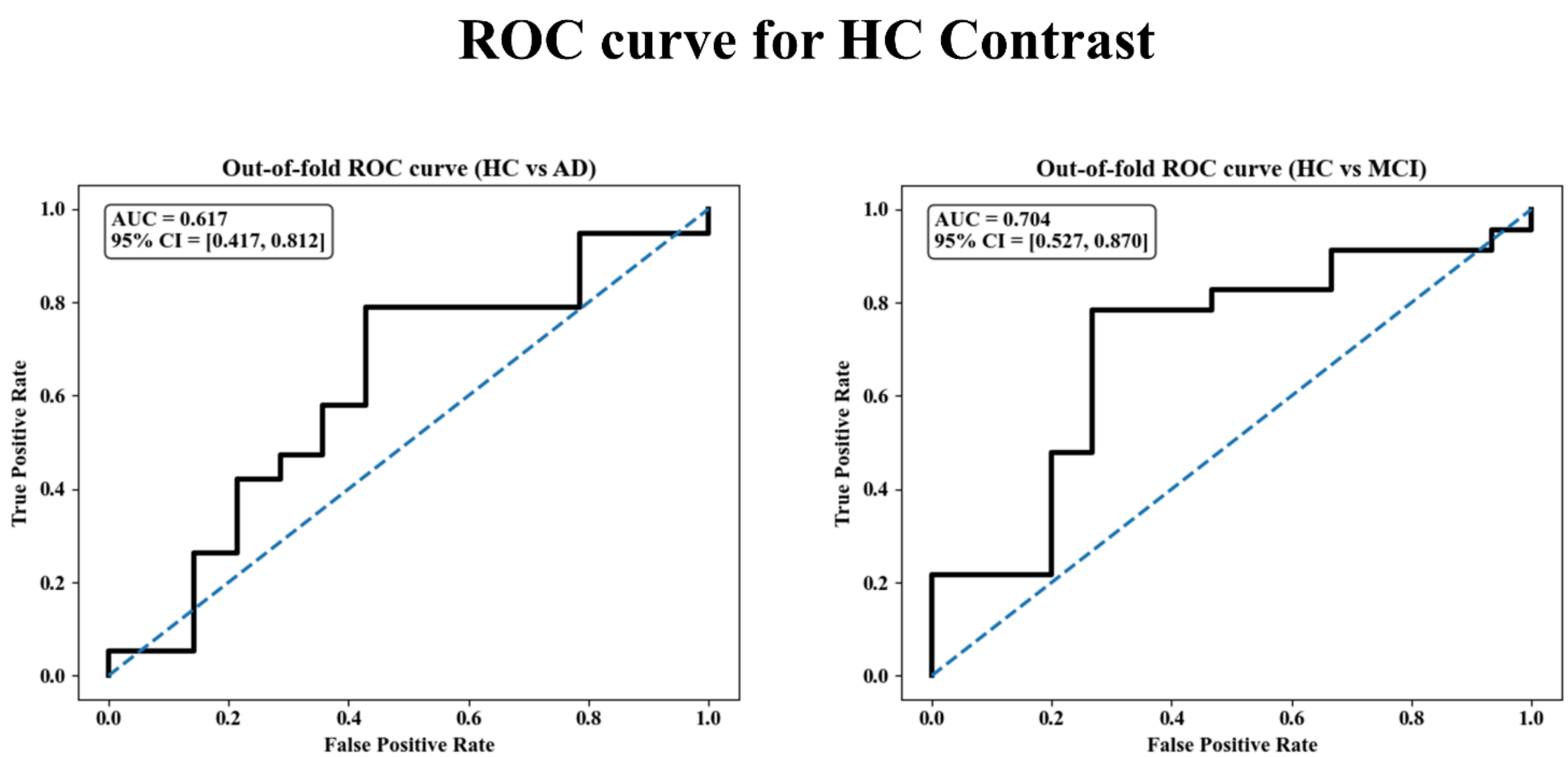

Supplementary Figure 4. Out-of-fold ROC curves for classification performance using HC as the reference group. Left: HC vs AD (AUC = 0.617, 95% CI: [0.417, 0.812]). Right: HC vs MCI (AUC = 0.704, 95% CI: [0.527, 0.870]). Solid black curves represent model performance, while the dashed diagonal line indicates chance-level classification. True positive rate (sensitivity) is plotted against false positive rate (1 − specificity).

1.2 Supplementary Tables

Supplementary Table 1. Edge-level statistics for all significant directed effective connectivity differences across group comparisons.

Task	Signal	Region-pair	β	q	95% CI
		**MCI vs AD**			
2-Back	HbR	DLPFC-L → FPC-R	-0.885	0.002	[-1.343, -0.391]
2-Back	HbR	DLPFC-R → FPC-R	-0.813	0.004	[-1.284, -0.305]
2-Back	HbR	FPC-L → FPC-R	-0.756	0.006	[-1.217, -0.243]
2-Back	HbR	OFC-L → FPC-R	-0.857	0.004	[-1.297, -0.386]
2-Back	HbR	OFC-R → FPC-R	-0.877	0.004	[-1.333, -0.394]
2-Back	HbR	VLPFC-L → FPC-R	-0.837	0.004	[-1.283, -0.343]
2-Back	HbR	VLPFC-R → FPC-R	-0.831	0.004	[-1.278, -0.322]
2-Back	HbO	DLPFC-R→ DLPFC-L	0.826	0.017	[0.371, 1.256]
2-Back	HbO	FPC-L → DLPFC-L	0.698	0.017	[0.178, 1.216]
2-Back	HbO	FPC-R→ DLPFC-L	0.613	0.025	[0.078, 1.140]
2-Back	HbO	OFC-L → DLPFC-L	0.659	0.019	[0.168, 1.117]
2-Back	HbO	OFC-R → DLPFC-L	0.835	0.017	[0.354, 1.286]
2-Back	HbO	VLPFC-L → DLPFC-L	0.759	0.017	[0.292, 1.179]
2-Back	HbO	VLPFC-R → DLPFC-L	0.669	0.019	[0.149, 1.146]
SVFT	HbR	DLPFC-L → FPC-R	-0.780	0.008	[-1.207, -0.379]

SVFT	HbR	DLPFC-R → FPC-R	-0.765	0.008	[-1.210, -0.346]
SVFT	HbR	FPC-L → FPC-R	-0.715	0.010	[-1.132, -0.302]
SVFT	HbR	OFC-L → FPC-R	-0.805	0.008	[-1.193, -0.437]
SVFT	HbR	OFC-R → FPC-R	-0.806	0.008	[-1.227, -0.409]
SVFT	HbR	VLPFC-L → FPC-R	-0.717	0.012	[-1.167, -0.340]
SVFT	HbR	VLPFC-R → FPC-R	-0.814	0.008	[-1.239, -0.398]
SVFT	HbO	DLPFC-R→ DLPFC-L	0.678	0.010	[0.250, 1.219]
SVFT	HbO	FPC-L → DLPFC-L	0.818	0.010	[0.401, 1.318]
SVFT	HbO	FPC-R→ DLPFC-L	0.672	0.010	[0.251, 1.167]
SVFT	HbO	OFC-L → DLPFC-L	0.658	0.011	[0.246, 1.137]
SVFT	HbO	OFC-R → DLPFC-L	0.732	0.010	[0.328, 1.241]
SVFT	HbO	VLPFC-L → DLPFC-L	0.720	0.010	[0.290, 1.294]
SVFT	HbO	VLPFC-R → DLPFC-L	0.703	0.010	[0.261, 1.209]
Stroop	HbR	DLPFC-R→ DLPFC-L	0.729	0.031	[0.257, 1.307]
Stroop	HbR	FPC-L → DLPFC-L	0.564	0.045	[0.104, 1.106]
Stroop	HbR	FPC-R→ DLPFC-L	0.675	0.036	[0.201, 1.209]
Stroop	HbR	OFC-L → DLPFC-L	0.636	0.036	[0.162, 1.186]
Stroop	HbR	OFC-R → DLPFC-L	0.643	0.036	[0.175, 1.154]
Stroop	HbR	VLPFC-R → DLPFC-L	0.854	0.030	[0.439, 1.373]
Stroop	HbR	DLPFC-L → FPC-L	0.732	0.014	[0.210, 1.302]
Stroop	HbR	DLPFC-R → FPC-L	0.834	0.007	[0.335, 1.399]
Stroop	HbR	FPC-R → FPC-L	0.828	0.007	[0.307 1.436]
Stroop	HbR	OFC-L → FPC-L	0.852	0.007	[0.329, 1.492]
Stroop	HbR	OFC-R → FPC-L	0.778	0.013	[0.235, 1.385]
Stroop	HbR	VLPFC-L → FPC-L	0.673	0.017	[0.114, 1.342]
Stroop	HbR	VLPFC-R → FPC-L	0.812	0.007	[0.292, 1.410]
Stroop	HbO	DLPFC-L → OFC-L	0.573	0.042	[0.030, 1.110]
Stroop	HbO	DLPFC-R → OFC-L	0.618	0.039	[0.128, 1.108]
Stroop	HbO	FPC-L → OFC-L	0.586	0.042	[0.069, 1.118]
Stroop	HbO	FPC-R → OFC-L	0.764	0.039	[0.241, 1.289]
Stroop	HbO	OFC-R → OFC-L	0.664	0.039	[0.166, 1.167]
Stroop	HbO	VLPFC-L → OFC-L	0.647	0.039	[0.136, 1.216]
Stroop	HbO	VLPFC-R → OFC-L	0.717	0.039	[0.230, 1.247]
Stroop	HbO	DLPFC-L → VLPFC-L	-0.688	0.038	[-1.331, -0.074]
Stroop	HbO	DLPFC-R → VLPFC-L	-0.718	0.038	[-1.340, -0.153]
Stroop	HbO	FPC-L → VLPFC-L	-0.621	0.038	[-1.302, -0.029]
Stroop	HbO	FPC-R → VLPFC-L	-0.557	0.038	[-1.264, 0.033]
Stroop	HbO	OFC-L → VLPFC-L	-0.617	0.038	[-1.269, -0.035]
Stroop	HbO	OFC-R → VLPFC-L	-0.594	0.038	[-1.208, -0.037]
Stroop	HbO	VLPFC-R → VLPFC-L	-0.532	0.047	[-1.175, 0.047]
		**HC vs AD**			
Stroop	HbO	DLPFC-L → OFC-L	0.983	0.044	[0.140, 1.855]
Stroop	HbO	DLPFC-R → OFC-L	0.964	0.044	[0.198, 1.777]
Stroop	HbO	FPC-L → OFC-L	0.905	0.044	[0.122, 1.927]
Stroop	HbO	FPC-R → OFC-L	0.916	0.044	[0.150, 1.845]
Stroop	HbO	OFC-R → OFC-L	1.084	0.044	[0.304, 1.922]
Stroop	HbO	VLPFC-R → OFC-L	0.833	0.045	[0.097, 1.688]
2-Back	HbR	DLPFC-L → FPC-R	-1.142	0.040	[-1.940, -0.185]

2-Back	HbR	FPC-L → FPC-R	-0.871	0.040	[-1.683, 0.054]
2-Back	HbR	OFC-L → FPC-R	-1.015	0.040	[-1.788, -0.070]
2-Back	HbR	OFC-R → FPC-R	-1.182	0.040	[-1.983, -0.330]
2-Back	HbR	VLPFC-L → FPC-R	-0.934	0.040	[-1.673, 0.025]
2-Back	HbR	VLPFC-R → FPC-R	-0.931	0.040	[-1.689, 0.049]
		**HC vs MCI**			
Rest	HbR	DLPFC-R→ DLPFC-L	0.965	0.010	[0.550, 1.357]
Rest	HbR	FPC-L → DLPFC-L	0.951	0.010	[0.501, 1.370]
Rest	HbR	FPC-R→ DLPFC-L	1.012	0.010	[0.543, 1.482]
Rest	HbR	OFC-L → DLPFC-L	0.899	0.010	[0.449, 1.361]
Rest	HbR	OFC-R → DLPFC-L	0.990	0.010	[0.526, 1.445]
Rest	HbR	VLPFC-L → DLPFC-L	0.910	0.010	[0.471, 1.363]
Rest	HbR	VLPFC-R → DLPFC-L	0.962	0.010	[0.482, 1.437]
2-Back	HbO	DLPFC-L→ VLPFC-R	-0.806	0.032	[-1.461, -0.219]
2-Back	HbO	DLPFC-R→ VLPFC-R	-0.866	0.029	[-1.479, -0.256]
2-Back	HbO	FPC-L → VLPFC-R	-0.899	0.029	[-1.486, -0.340]
2-Back	HbO	FPC-R→ VLPFC-R	-0.880	0.029	[-1.528, -0.235]
2-Back	HbO	OFC-L → VLPFC-R	-1.088	0.029	[-1.685, -0.475]
2-Back	HbO	OFC-R → VLPFC-R	-0.904	0.029	[-1.541, -0.255]
2-Back	HbO	VLPFC-L → VLPFC-R	-0.776	0.032	[-1.406, -0.177]

This table reports all significant directed effective connectivity differences identified using transfer entropy (TE) analysis across group comparisons (HC vs MCI, HC vs AD, and MCI vs AD). For each task and signal type, a region pair denotes the directed connection from the source to the target region. β represents the standardized effect size obtained from linear mixed-effects models (LMMs), where positive values indicate increased connectivity in the second group relative to the first, and negative values indicate decreased connectivity. q-values correspond to false discovery rate (FDR)-corrected p-values across all tested connections. The 95% confidence intervals (CI) were estimated using bootstrap resampling. Only connections surviving multiple comparison correction are included. Abbreviations: DLPFC, dorsolateral prefrontal cortex; FPC, frontopolar cortex; OFC, orbitofrontal cortex; VLPFC, ventrolateral prefrontal cortex; HbO, oxygenated hemoglobin; HbR, deoxygenated hemoglobin; SVFT, semantic verbal fluency task.

Supplementary Table 2. Sensitivity analysis of significant effective connectivity patterns using k = 4.

Target	Task	Signal	No. of Robust primary Significant Edges	Robust	Attenuated	Unstable
**MCI vs AD**						
FPC-R	2-Back	HbR	7	7	0	0
FPC-R	SVFT	HbR	7	7	0	0
DLPFC-L	2-Back	HbO	7	7	0	0
DLPFC-L	SVFT	HbO	7	7	0	0
DLPFC-L	Stroop	HbR	6	6	0	0
FPC-L	Stroop	HbR	7	7	0	0
OFC-L	Stroop	HbO	7	4	0	3
VLPFC-L	Stroop	HbO	5	0	0	5
**HC vs AD**						
OFC-L	Stroop	HbO	6	5	0	1
FPC-R	2-Back	HbR	3	1	0	2
**HC vs MCI**						
DLPFC-L	Rest	HbR	7	7	0	0
VLPFC-R	2-Back	HbO	7	7	0	0

This table summarizes the robustness of significant connections identified in the primary analysis under k = 4 (Kraskov estimator). “No. of Robust primary Significant Edges” indicates the total number of significant edges per target. “Robust” denotes edges remaining significant with consistent direction, “Attenuated” denotes edges with reduced effect size but preserved direction, and “Unstable” denotes edges losing significance or changing direction. Abbreviations: HbO, oxygenated hemoglobin; HbR, deoxygenated hemoglobin; SVFT, semantic verbal fluency task.

Supplementary Table 3. Sensitivity analysis of significant effective connectivity patterns without including age as a covariate

Target	Task	Signal	No. of Robust Primary Significant Edges	Robust	Attenuated	Fragile
**MCI vs AD**						
FPC-R	2-Back	HbR	7	7	0	0
FPC-R	SVFT	HbR	7	7	0	0
DLPFC-L	2-Back	HbO	7	7	0	0
DLPFC-L	SVFT	HbO	7	7	0	0
DLPFC-L	Stroop	HbR	6	5	0	1
FPC-L	Stroop	HbR	7	6	0	1
OFC-L	Stroop	HbO	7	4	0	3
VLPFC-L	Stroop	HbO	5	2	0	3
**HC vs AD**						
OFC-L	Stroop	HbO	6	0	0	6
FPC-R	2-Back	HbR	3	0	0	3
**HC vs MCI**						
DLPFC-L	Rest	HbR	7	0	7	0
VLPFC-R	2-Back	HbO	7	0	1	6

This table summarizes the robustness of significant connections identified in the primary analysis after excluding age as a covariate in the linear mixed-effects models. “No. of Robust Primary Significant Edges” indicates the total number of significant edges per target. “Robust” denotes edges remaining significant with consistent direction, “Attenuated” denotes edges with reduced effect size but preserved direction, and “Fragile” denotes edges losing significance or showing inconsistent direction. Abbreviations: HbO, oxygenated hemoglobin; HbR, deoxygenated hemoglobin; SVFT, semantic verbal fluency task.

Supplementary Table 4. Sensitivity analysis of significant effective connectivity patterns without including sex as a covariate.

Target	Task	Signal	No. of Robust Primary Significant Edges	Robust	Attenuated	Fragile
			**MCI vs AD**			
FPC-R	2-Back	HbR	7	7	0	0
FPC-R	SVFT	HbR	7	7	0	0
DLPFC-L	2-Back	HbO	7	7	0	0
DLPFC-L	SVFT	HbO	7	7	0	0
DLPFC-L	Stroop	HbR	6	6	0	0
FPC-L	Stroop	HbR	7	7	0	0
OFC-L	Stroop	HbO	7	7	0	0
VLPFC-L	Stroop	HbO	5	5	0	0
			**HC vs AD**			
OFC-L	Stroop	HbO	6	6	0	0
FPC-R	2-Back	HbR	3	3	0	0
			**HC vs MCI**			
DLPFC-L	Rest	HbR	7	7	0	0
VLPFC-R	2-Back	HbO	7	7	0	0

This table summarizes the robustness of significant connections identified in the primary analysis after excluding sex as a covariate in the linear mixed-effects models. "No. of Robust primary Significant Edges" indicates the total number of significant edges per target. "Robust" denotes edges remaining significant with consistent direction, "Attenuated" denotes edges with reduced effect size but preserved direction, and "Fragile" denotes edges losing significance or showing inconsistent direction. Abbreviations: HbO, oxygenated hemoglobin; HbR, deoxygenated hemoglobin; SVFT, semantic verbal fluency task.

Supplementary Table 5. Sensitivity analysis of significant effective connectivity patterns using surrogate-normalized transfer entropy.

Target	Task	Signal	Stable	Attenuated	Partially Stable	Unstable
			**MCI vs AD**			
FPC-R	2-Back	HbR	Yes	No	No	No
FPC-R	SVFT	HbR	No	No	No	Yes
DLPFC-L	2-Back	HbO	No	No	Yes	No
DLPFC-L	SVFT	HbO	No	No	Yes	No
DLPFC-L	Stroop	HbR	No	No	No	Yes
FPC-L	Stroop	HbR	No	No	Yes	No
OFC-L	Stroop	HbO	No	No	Yes	No
VLPFC-L	Stroop	HbO	No	No	Yes	No
			**HC vs AD**			
OFC-L	Stroop	HbO	No	Yes	No	No
FPC-R	2-Back	HbR	No	Yes	No	No
			**HC vs MCI**			
DLPFC-L	Rest	HbR	No	No	Yes	No
VLPFC-R	2-Back	HbO	No	No	Yes	No

This table summarizes the stability of significant connections identified in the primary analysis after applying surrogate-normalized transfer entropy (TE). "Stable" indicates effects that remained significant with consistent direction, "Attenuated" indicates reduced effect magnitude with preserved direction, "Partially Stable" indicates effect size direction preserved but CI crosses zero, and $p > 0.1$, and "Unstable" indicates effect size direction reversed along with CI crosses zero and $p > 0.1$. Classifications are based on comparison with primary (non-surrogate) results. Abbreviations: HbO, oxygenated hemoglobin; HbR, deoxygenated hemoglobin; SVFT, semantic verbal fluency task.
